# Combinatorial Measurement of *CDKN1A*/*p21* and *KIF20A* Expression for Discrimination of DNA Damage-Induced Clastogenicity

**DOI:** 10.3390/ijms151017256

**Published:** 2014-09-26

**Authors:** Rina Sakai, Yuji Morikawa, Chiaki Kondo, Hiroyuki Oka, Hirofumi Miyajima, Kihei Kubo, Takeki Uehara

**Affiliations:** 1 Developmental Research Laboratories, Shionogi & Co., Ltd., 3-1-1 Futaba-cho, Toyonaka, Osaka 561-0825, Japan; E-Mails: rina.sakai@shionogi.co.jp (R.S.); yuji.morikawa@shionogi.co.jp (Y.M.); chiaki.kondou@shionogi.co.jp (C.K.); hiroyuki.oka@shionogi.co.jp (H.O.); hirofumi.miyajima@shionogi.co.jp (H.M.); 2 Department of Veterinary Science, Graduate School of Life and Environmental Sciences, Osaka Prefecture University, 1-58 Rinkuu Ourai Kita, Izumisano, Osaka 598-8531, Japan; E-Mail: kubokrad@vet.osakafu-u.ac.jp

**Keywords:** clastogenicity, DNA damage, kinesin family member 20A (*KIF20A*), cyclin-dependent kinase inhibitor 1A (*CDKN1A*)/*p21*, TK6 lymphoblastoid cell line, toxicogenomics

## Abstract

*In vitro* mammalian cytogenetic tests detect chromosomal aberrations and are used for testing the genotoxicity of compounds. This study aimed to identify a supportive genomic biomarker could minimize the risk of misjudgments and aid appropriate decision making in genotoxicity testing. Human lymphoblastoid TK6 cells were treated with each of six DNA damage-inducing genotoxins (clastogens) or two genotoxins that do not cause DNA damage. Cells were exposed to each compound for 4 h, and gene expression was comprehensively examined using Affymetrix U133A microarrays. Toxicogenomic analysis revealed characteristic alterations in the expression of genes included in *cyclin-dependent kinase inhibitor 1A* (*CDKN1A/p21*)-centered network. The majority of genes included in this network were upregulated on treatment with DNA damage-inducing clastogens. The network, however, also included *kinesin family member 20A* (*KIF20A*) downregulated by treatment with all the DNA damage-inducing clastogens. Downregulation of *KIF20A* expression was successfully confirmed using additional DNA damage-inducing clastogens. Our analysis also demonstrated that nucleic acid constituents falsely downregulated the expression of *KIF20A*, possibly via *p16* activation, independently of the *CDKN1A* signaling pathway. Our results indicate the potential of *KIF20A* as a supportive biomarker for clastogenicity judgment and possible mechanisms involved in *KIF20A* downregulation in DNA damage and non-DNA damage signaling networks.

## 1. Introduction

*In vitro* mammalian cytogenetic tests detect chromosomal aberrations in cultured mammalian cells, and are, thus, used for testing the genotoxicity of compounds. However, false positive results associated with excessive toxicity frequently occur in these tests [1]. Therefore, there is a growing demand for mechanism-based follow-up assays to confirm positive results from cytogenetic tests. In a previous study [2], we applied a toxicogenomic approach to establish a classification tool based on clastogenic mechanisms. Human lymphoblastoid TK6 cells were treated with each of eight different genotoxins, including six DNA damage-inducing genotoxins (clastogens) and two genotoxins that do not cause DNA damage. The following six DNA damage-inducing clastogens were included: two cross-linking compounds (mitomycin C: MMC and cisplatin: CP) and alkylating compounds (methyl methanesulfonate: MMS and ethyl methanesulfonate: EMS), a topoisomerase II inhibitor (etoposide: ETOP), and an antimetabolite (hydroxyurea: HU) targeting DNA synthesis. The following genotoxins that do not induce DNA damage were included: a mitotic spindle inhibitor (colchicine: COLCH) and a DNA nucleoside (adenine: ADE) that induces chromosomal aberrations in a secondary manner. Cells were exposed to each compound for 4 h, and gene expression was comprehensively examined using Affymetrix U133A microarrays. Toxicogenomics data analysis identified cyclin-dependent kinase inhibitor 1A (*CDKN1A*)-centered interactome as the most significant network, based on significantly-altered discriminative genes of DNA damage; moreover, *CDKN1A* was reported the top-ranked gene for correct classification. Our previous study also suggested that *CDKN1A* could be used as a biomarker for discriminating chromosomal aberrations that result from DNA damage from other types of chromosomal aberrations. Therefore, *CDKN1A* expression in TK6 cells could be used as a follow-up assay for validating positive results from mammalian cytogenetic tests. However, we need to pay attention during the actual decision-making process because *CDKN1A* expression is affected by the cytotoxic condition, as shown in the previous report. Mechanistically, *CDKN1A* expression is tightly controlled by the DNA damage-responsive gene *p53*, and upregulation of *CDKN1A* is reported to be affected by various factors, such as apoptosis [3], antioxidative effects [4], and a number of transcription factors [5]. Moreover, in the previous study, *CDKN1A* upregulation was also observed on treatment with several compounds that do not cause DNA damage; however, the extent of upregulation was lower than the optimal threshold. Identification of optimal doses and cell sampling conditions is therefore required for minimizing the risk of misclassification when applying *CDKN1A* as a biomarker for discriminating chromosomal aberrations. Further efforts are also warranted for minimizing risk that could lead to misjudgments, particularly when the application of this tool across multiple facilities is considered. In the present study, we hypothesized that a combinatorial measurement of the expression of multiple genes could minimize the risk of misjudgments and aid appropriate decision making. Although the previous study focused only on *CDKN1A* as the top-ranked among all genes with significantly altered expression, several characteristic alterations were observed in the expression of genes included in *CDKN1A*-centered network. The current study highlights the potential of downregulated genes as supportive biomarkers for interpreting the biological significance of *CDKN1A* upregulation and for facilitating correct classification.

## 2. Results and Discussion

### 2.1. Gene Expression Profile of a Newly Selected Candidate Biomarker, KIF20A

**Figure 1:**
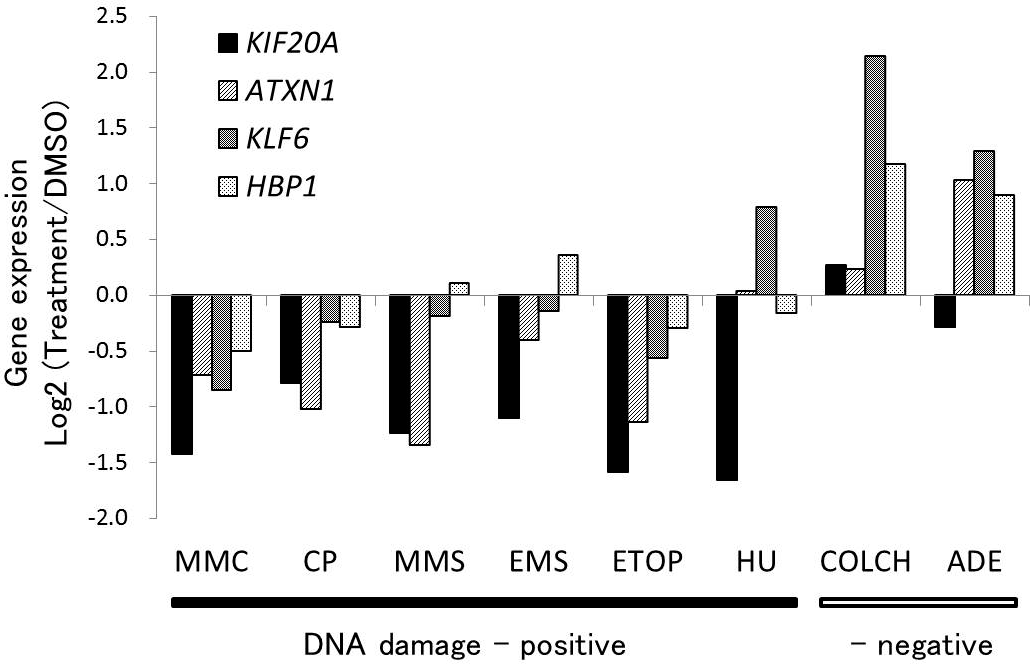
Expression profiles of four genes on treatment with the genotoxins used in microarray analysis. The genes *ATXN1*, *KIF20A*, *KLF6*, and *HBP1*, which are a part of *CDKN1A*-centered network, were downregulated in response to DNA damage. The log base 2 values of the ratio of alterations in the expression of these four downregulated genes to the mean values of the corresponding control obtained from the microarray data (single experiment in duplicate) are shown. TK6 cells were treated with MMC, mitomycin C; CP, cisplatin; MMS, methyl methanesulfonate; EMS, ethyl methanesulfonate; ETOP, etoposide; HU, hydroxyurea; COLCH, colchicine; and ADE, adenine; for 4 h at concentrations corresponding to 50% of relative cell growth (RCG) compared to number of cells in vehicle control, and allowed to recover for 20 h in standard cell culture medium.

The Human HG-U133A DNA microarray, which contains 22,000 probes, was used for performing a comprehensive analysis of gene expression in TK6 cells, individually treated with the eight genotoxins, including six DNA damage-inducing genotoxins (clastogens) and two genotoxins that do not cause DNA damage, as shown previously [2]. Of the genes involved in *CDKN1A*-centered network, which was revealed as the most significant network by a functional network analysis [2], four genes, *ataxin 1* (*ATXN1*), *KIF20A*, *Kruppel-like factor 6* (*KLF6*), and *HMG-box transcription factor 1* (*HBP1*), were downregulated on treatment with DNA damage-inducing compounds. Figure 1 shows the log base 2 values of the ratio of alterations in the expression of these four genes to the means of the corresponding control. The only gene that showed consistent downregulation on treatment with all the DNA damage-inducing clastogens was *KIF20A*.

To validate the potential of *KIF20A* as a biomarker for clastogenic damage, TK6 cells were treated with nine test compounds, including four DNA damage-inducing clastogens (an alkylating compound, *N*-ethyl-*N*-nitrosourea: ENU; a cross-linking compound, 1-(2-chloroethyl)-3-cyclohexyl-1-nitrosourea: CCNU; a topoisomerase I inhibitor, camptothecin: CAMP; and an antimetabolite targeting DNA synthesis, 5-fluorouracil: 5-FU) and five genotoxins that do not induce DNA damage (mitotic spindle inhibitors, paclitaxel: TXL and colcemid: COLCE; a DNA precursor, 2-deoxyadenosine: 2-DA; an inhibitor of protein synthesis, cycloheximide: CHX; and a compound that affects osmolality, sodium chloride: NaCl). Furthermore, three newly-synthesized drug candidates (compounds A, B, and C), which induced 10% (positive), 0% (negative), and 2% (negative) chromosomal aberrations to the cells, respectively, were also used as test compounds. In the *in vitro* chromosomal aberration test, compounds for which ≥10% of the cells were aberrant were categorized as positive. In addition to these twelve test compounds, compounds examined by microarray analysis were also assessed by quantitative RT-PCR analysis (QPCR).

**Table 1:** A list of compounds used in the validation study.

DNA Damage Class	Compounds ^a^	Mechanism	Concentration (μg/mL)	RCG ^b^ (%)
Positive	MMC	Cross-link [6]	1	47.5
	CP	Cross-link [7]	10	52.7
	MMS	Alkylate [8]	40	42.9
	EMS	Alkylate [8]	1200	47.8
	ETOP	Topoisomerase II inhibitor [9]	1	40.5
	HU	Antimetabolite in DNA synthesis [10]	2500	41.3
	ENU	Alkylate [8]	125	44.6
	CCNU	Cross-link [11]	10	54.4
	CAMP	Topoisomerase I inhibitor [12]	0.005	57.0
	5-FU	Antimetabolite in DNA synthesis [13]	25	46.0
	A	Positive in the CA test ^c^	204	50.3
Negative	COLCH	Mitotic spindle inhibitor [14]	0.04	42.7
	ADE	DNA precursor [15]	1400	44.2
	TXL	Mitotic spindle inhibitor [16]	0.25	54.5
	COLCE	Mitotic spindle inhibitor [17]	10	37.9
	2-DA	DNA precursor [15]	4000	58.5
	CHX	Inhibitor of protein synthesis [18]	800	52.3
	NaCl ^d^	Osmolality change [19]	0.80%	47.1
	B	Negative in the CA test ^c^	346	48.5
	C	Negative in the CA test ^c^	346	49.8

^a^ MMC, mitomycin C; CP, cisplatin; MMS, methyl methanesulfonate; EMS, ethyl methanesulfonate; ETOP, etoposide; HU, hydroxyurea; ENU, *N*-ethyl-*N*-nitrosourea; CCNU, 1-(2-chloroethyl)-3-cyclohexyl-1-nitrosourea; CAMP, camptothecin; 5-FU, 5-fluorouracil; COLCH, colchicines; ADE, adenine; TXL, paclitaxel; COLCE, colcemid; 2-DA, 2-deoxyadenosine; CHX, cycloheximide; NaCl, sodium chloride; A, B, C, newly-synthesized compounds. All compound solutions contain DMSO (1% *v*/*v*); ^b^ RCG, relative cell growth indicated as relative number of cells compared to vehicle control; ^c^ CA test, *In vitro* chromosomal aberration test; and ^d^ 10% (*v*/*v*) NaCl was used. Sterilized water was used as vehicle control.

**Figure 2:**
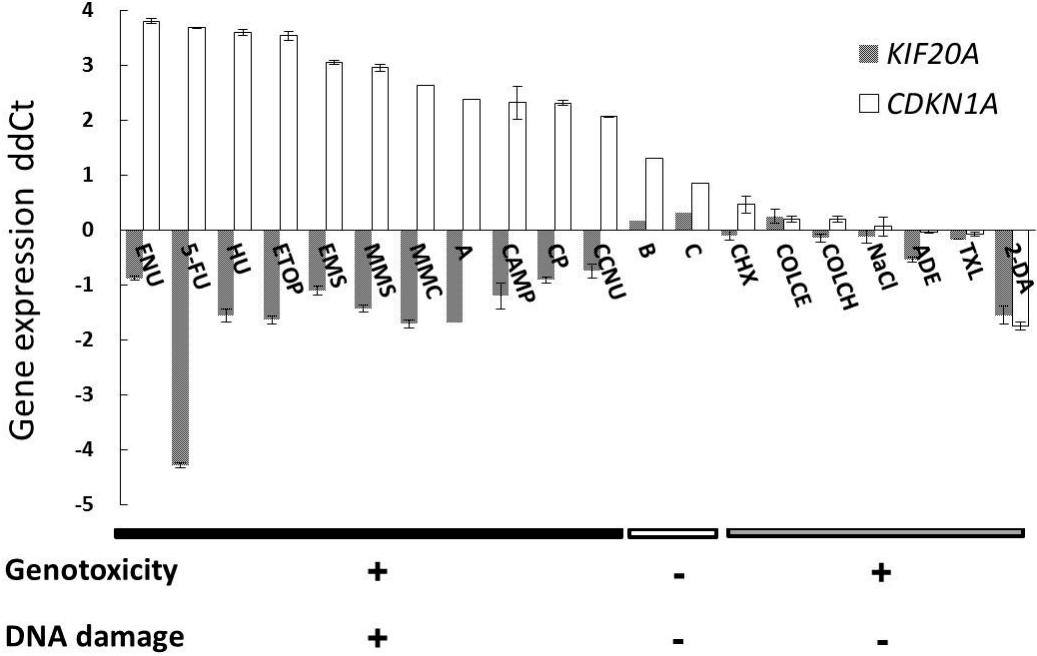
Validation of expression alterations in *KIF20A* in parallel with *CDKN1A.* Quantitative RT-PCR for analysis of gene expression alterations induced in TK6 cells after treatment for 4 h with three groups of compounds: (i) DNA damage-inducing genotoxins (clastogens); (ii) compounds that do not induce chromosomal aberrations; and (iii) genotoxins that do not involve DNA damage. Compound group (i) includes ENU, *N*-ethyl-*N*-nitrosourea; 5-FU, 5-fluorouracil; HU, hydroxyurea; ETOP, etoposide; EMS, ethyl methanesulfonate; MMS, methyl methanesulfonate; MMC, mitomycin C; CAMP, camptothecin; CP, cisplatin; CCNU, 1-(2-chloroethyl)-3-cyclohexyl-1-nitrosourea; and a newly-synthesized drug candidate (designated A) positive in the *in vitro* chromosomal aberration test. Compound group (ii) includes two newly-synthesized drug candidates (designated B and C) negative in the *in vitro* chromosomal aberration test. Compound group (iii) includes CHX, cycloheximide; COLCE, colcemid; COLCH, colchicines; NaCl, sodium chloride; ADE, adenine; TXL, paclitaxel; and 2-DA, 2-deoxyadenosine. The means ± standard deviation (SD) of cycle threshold (*C*t) values were shown (single experiment in duplicate).

QPCR was used for examining alterations in *KIF20A* expression; the treatment concentrations were set at approximately 50% of relative cell growth (RCG50) compared to number of cells in vehicle control (Table 1), and shown in parallel with *CDKN1A* (Figure 2). *KIF20A* was downregulated in TK6 cells treated with all the DNA damage-inducing clastogens, including compound A; however, it was also downregulated by nucleic acid constituents such as ADE and 2-DA that do not induce DNA damage. Treatment with the compounds B and C, which are negative for clastogenicity, did not alter *KIF20A* expression, while *CDKN1A* expression was moderately upregulated (dd*C*_t_ = 1.30 and 0.85, respectively).

### 2.2. Expression Changes of Retinoblastoma (RB)/E2 Promoter Binding Factor (E2F) Regulatory Genes

The retinoblastoma (RB)/E2 promoter binding factor (E2F) pathway plays a key role in cell cycle progression from G1 to S phases, and also regulates the G2/M checkpoint [20,21]. As a result, RB-defective cells do not undergo G2/M arrest in response to a DNA damage-inducing stimulus [20], while the ectopic overexpression of E2F family proteins induces many genes involved in mitosis [21]. *CDKN1A*, which is DNA-damage responsive, is a potent cyclin-dependent kinase inhibitor which binds to and inhibits cyclin/CDK2 or CDK4 complexes [22], and, thus, acts as a regulator of cell cycle progression through RB/E2F pathway [23].

To clarify the relation of RB/E2F pathway and *KIF20A* expression change, microarray data of RB/E2F regulatory G2/M-related eight genes, *CENPE*, *CDC25C*, *CDCA8*, *KIF11*, *ECT2*, *NEK2*, *CCNA2*, *KIF18A* [20], were shown in Table 2. As a result, RB/E2F regulatory genes related to G2/M cell cycle were downregulated by all the DNA-damaging clastogens (MMC, MMS, ETOP, HU, EMS, and CP). This result suggests that RB/E2F pathway is downregulated in response to DNA damage, leading to G2/M mitotic cell cycle arrest.

**Table 2:** Expression change of RB/E2F regulatory genes related to G2/M cell cycle.

Gene Symbol	Probe Set ID	log2 (MMC/D)	log2 (MMS/D)	log2 (ETOP/D)	log2 (HU/D)	log2 (EMS/D)	log2 (CP/D)	log2 (ADE/D)
*CCNA2*	203418_at	−0.345	−0.463	−0.602	−0.635	−0.680	−0.305	−0.771
*CCNA2*	213226_at	−0.358	−0.334	−0.671	−0.602	−0.429	−0.221	−0.540
*CDC25C*	205167_s_at	−0.961	−0.403	−0.696	−0.657	−0.354	−0.735	−1.963
*CDC25C*	217010_s_at	−0.506	−0.411	−0.615	−0.402	−0.597	−0.509	−1.454
*CDCA8*	221520_s_at	−0.653	−0.415	−0.930	−0.728	−0.498	−0.547	−1.130
*CENPE*	205046_at	−1.389	−1.107	−1.968	−1.414	−0.917	−0.637	−1.610
*ECT2*	219787_s_at	−0.607	−0.651	−1.033	−0.881	−0.574	−0.642	−1.526
*KIF11*	204444_at	−0.486	−0.325	−0.729	−0.035	−0.349	−0.409	−0.951
*KIF18A*	221258_s_at	−1.037	−0.571	−1.125	−0.395	−0.750	−0.873	−2.083
*NEK2*	204641_at	−0.832	−0.500	−1.186	−0.861	−0.436	−0.394	−0.602
*NEK2*	211080_s_at	−0.780	−0.881	−1.230	−0.948	−0.647	−0.400	−1.668

Shown are the log base 2 value of the ratio of alternations in the expression of eight genes to the mean of the corresponding control, DMSO. MMC, mitomycin C; MMS, methyl methanesulfonate; ETOP, etoposide, HU, hydroxyurea; EMS, ethyl methanesulfonate; CP, cisplatin; and ADE, adenine.

*KIF20A* has been reported to strongly bind E2F [24], and its expression is regulated by E2F [25]. KIF20A has been reported to be a microtubule-associated motor protein, essential for chromosome segregation and mitosis [26] and for integrating cell cycle progression with the G2/M checkpoint [27]. Taken together, the evidence suggests that *KIF20A* downregulation is involved in the downregulation of RB/E2F pathway as a downstream signal of *CDKN1A* upregulation, resulting in G2/M checkpoint activation in response to DNA damage. Furthermore, these DNA damage-related genes would serve as key biomarkers for the discrimination of DNA damage-inducing clastogenicity.

For the actual application of the newly-identified biomarker, *KIF20A*, as a supporting tool for the discrimination of DNA damage-inducing clastogenicity, we examined the compounds B and C, which tested negative in the chromosomal aberration test. These two compounds resulted in moderate *CDKN1A* upregulation (dd*C*_t_ = 1.30 and 0.85, respectively) but not *KIF20A* downregulation. DNA double strand breaks, if left unrepaired, could lead to clastogenicity [28], and the resulting DNA imperfections induced by DNA damage-inducing clastogens could activate the G2/M checkpoint. This, in turn, suggested that the compounds B and C (which were negative for chromosomal aberration) did not induce G2/M arrest. Taken together, the data strongly supports the notion that *KIF20A* downregulation in relation to the G2/M checkpoint greatly enhances the ability for discrimination based on DNA damage-responsible *CDKN1A*.

### 2.3. Biological Significance of Genes Differentially Regulated on Adenine (ADE) Treatment

*KIF20A* was downregulated on treatment with not only DNA damage-inducing clastogens but also nucleic acid constituents, including ADE and 2-DA (Figure 2). The eight RB/E2F regulatory genes related to cell cycle [20] were also downregulated on treatments with ADE, as well as DNA damage-inducing clastogens (Table 2).

**Figure 3:**
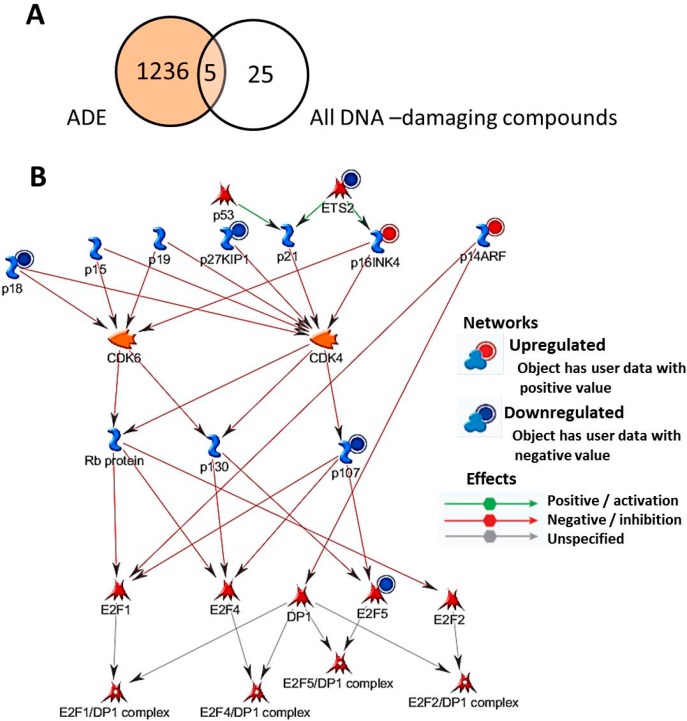
Pathway enrichment analysis for toxicity networks showing E2F downregulation. Venn diagram (**A**) showing intersection of gene sets. Five of 1241 probes were altered in common on treatments with adenine (ADE) and DNA damage-inducing clastogens, while 1236 probes were specifically altered on ADE treatment. The network comprising the regulation of cyclin-dependent kinase (CDK) 4, 6 by CDK inhibitors (**B**) was obtained by pathway enrichment analysis showing E2F downregulation using the 1236 ADE-induced genes depicted in (**A**) by MetaCore.

To clarify the mechanism of *KIF20A* downregulation on treatment with nucleic acid constituents, microarray data obtained from cells treated with ADE was compared with data obtained with the other DNA damage-inducing clastogens, namely, MMC, CP, MMS, EMS, ETOP, and HU. By applying statistical criteria for gene selection, 1241 probes were selected as differentially regulated genes on treatment with ADE, while 1409, 1357, 2088, 1891, 1726, or 2647 probes represented differentially regulated genes on treatment with MMC, CP, MMS, EMS, ETOP, or HU, respectively; 30 probes showed expression alters with all DNA damage-inducing clastogens. Figure 3A shows the Venn diagram depicting the selected probes on treatments with ADE and DNA damage-inducing clastogens, and the intersection of these probes. Five of the 1241 probes were altered by ADE, as well as DNA damage-inducing clastogens, and 1236 probes were specifically altered on treatment with ADE (Table S1).

The biological significance of alterations in these 1236 probes on treatment with ADE was characterized. Gene ontology (GO) analysis on these 1236 probes revealed that genes involved in RNA metabolic processes and the M-phase of the mitotic cell cycle were enriched in the probes specifically altered by ADE treatment (Table 3). To determine the possible signaling pathways leading to *KIF20A* downregulation, network analysis was performed on these 1236 probes that were specifically altered on treatment with ADE. Among the significant networks identified in the analysis, two networks, including “anaphase-promoting complex (APC) regulation of G1/S” and “cyclin-dependent kinase (CDK) 4, 6 regulation by cyclin/CDK inhibitors” were shown to be linked with E2F downregulation. Figure 3B illustrates one of the identified networks, cell cycle control via CDK4, 6 regulation by CDK inhibitors, and indicates that activation of *p16* results in inhibition of E2Fs through CDK4, CDK6, and retinoblastoma (RB) family proteins (p130, p107, and RB), independently of *CDKN1A*/*p21*.

**Table 3:** Gene ontology analysis for 1236 probes selected from ADE-treated TK6 cells.

Gene Ontology ID	Biological Processes	*p*-Value (Benjamini)
GO:0016070	RNA metabolic process	6.15 × 10^−17^
GO:0006396	RNA processing	4.28 × 10^−13^
GO:0000279	M phase	6.26 × 10^−8^
GO:0007067	Mitosis	7.05 × 10^−8^
GO:0019219	Regulation of nucleobase, nucleoside, nucleotide and nucleic acid metabolic process	8.70 × 10^−8^
GO:0010468	Regulation of gene expression	7.34 × 10^−8^
GO:0000087	M phase of mitotic cell cycle	7.16 × 10^−8^
GO:0010556	Regulation of macromolecule biosynthetic process	9.86 × 10^−8^
GO:0045449	Regulation of transcription	1.45 × 10^−7^
GO:0031326	Regulation of cellular biosynthetic process	1.67 × 10^−6^
GO:0034470	ncRNA processing	2.09 × 10^−5^
GO:0016071	mRNA metabolic process	4.97 × 10^−5^
GO:0008380	RNA splicing	1.14 × 10^−4^
GO:0006397	mRNA processing	1.63 × 10^−4^

The functional annotation clustering tool DAVID (Database for Annotation, Visualization, and Integrated Discovery) was used for gene ontology (GO) analysis (*p* < 0.001).

**Figure 4:**
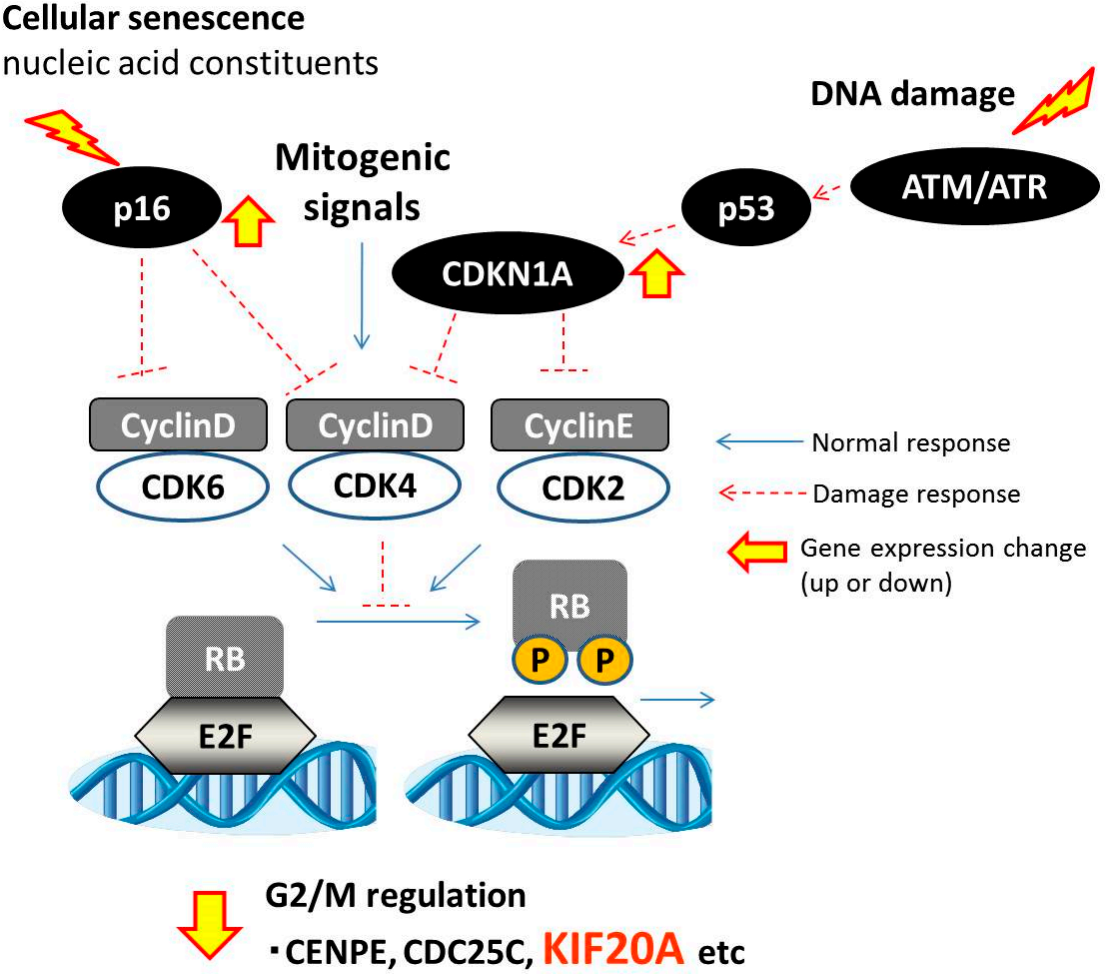
Proposed model of the roles of *CDKN1A* and *KIF20A* in cell cycle control via RB/E2F pathway. Mitogenic signals induce cell cycle progression, regulating cyclin D/cyclin-dependent kinase (CDK) 4 or 6, and cyclin E/CDK2, and resulting in retinoblastoma (RB) phosphorylation. Phosphorylated RB releases E2F transcription factor, resulting in the induction of E2F transcriptionaltarget genes including *KIF20A*. DNA damage induces upregulation of *CDKN1A* and downregulation of *KIF20A* via the RB/E2F pathway. Nucleic acid constituents induce downregulation of *KIF20A* by activation of *p16* via RB/E2F; this pathway is termed cellular senescence response.

Figure 4 illustrates cell cycle control via the RB/E2F pathway induced by either DNA damage-inducing clastogens, which caused downregulation of *KIF20A* via activation of *CDKN1A*, or by nucleic acid constituents (ADE) via *p16* (cyclin-dependent kinase inhibitor 2A) activation, independently of *CDKN1A*. The *p16*-regulated RB/E2F pathway is reported as the cellular senescence pathway [29], which is downregulated by a variety of extrinsic physiological stresses, oncogene activation, and toxicity from oxygen free radicals [30]. Because certain compounds downregulate *KIF20A* expression independently of DNA damage, discrimination based on *KIF20A* expression must be cautiously conducted. In addition, although the present study only focused on alterations of transcriptional regulation of genes in the response to DNA damage, there is growing evidence that epigenetic changes play a critical role in the regulation of expression of genes including *CDKN1A* [31]. Consequently, further research is warranted regarding the involvement of epigenetic factors can affect the *CDKN1A* and *KIF20A* expression in the response to DNA damage.

## 3. Experimental Section

### 3.1. In Vitro Cell Culture and Treatment

In the present study, microarray data and total RNA samples (for RT-PCR analysis) obtained previously [2] were used. Briefly, the human B lymphoblastoid cell line TK6 (DS Pharma Biomedical Co., Ltd., Osaka, Japan) was grown in suspension in RPMI 1640 medium (Sigma Aldrich, St. Louis, MO, USA) supplemented with 10% (*v*/*v*) heat-inactivated horse serum (Gibco Invitrogen Corporation, Grand island, NY, USA), 100 U/mL of penicillin, and 100 μg/mL of streptomycin (Sigma Aldrich). Cells were maintained at 37 °C in a humidified atmosphere of 5% CO_2_. Exponentially growing cultures (at cell density of 5 × 10^5^ cells/mL) were treated with the compounds. MMS, EMS, ETOP, HU, COLCH, ADE, ENU, CCNU, 5-FU, CAMP, TXL, 2-DA, and CHX were purchased from Sigma Aldrich. MMC, CP, and COLCE were purchased from Kyowa Hakko Kirin Co., Ltd. (Tokyo, Japan), Nippon Kayaku Co., Ltd. (Tokyo, Japan), and Wako Pure Chemical Industries, Ltd. (Osaka, Japan), respectively. Compounds A, B, and C were synthesized as drug candidates by Shionogi & Co., Ltd. (Osaka, Japan). These compounds were dissolved in dimethyl sulfoxide (DMSO; Wako Pure Chemical Industries, Ltd.) at a final concentration of 1% (*v*/*v*) in cell culture medium. NaCl was purchased from Wako Pure Chemical Industries, Ltd., dissolved in sterilized water and filtered prior to use. All test solutions were prepared immediately prior to use.

After treatment with the compounds for 4 h, a fraction of cells were lysed in RLT buffer (RNeasy Mini Kit, Qiagen, Valencia, CA, USA) and collected for gene expression analysis. The cytotoxicity of the compounds was evaluated by means of relative cell growth (RCG). For this purpose, the remaining cells were washed with PBS and allowed to recover for 20 h in standard cell culture medium. For microarray and quantitative RT-PCR analyses, cells were treated with particular concentrations of each compound, which resulted in approximately 50% reduction in RCG (RCG50). As *CDKN1A* expression was reported to be a biomarker for DNA damage-inducing clastogenicity under RCG50 [2], the concentration indicated RCG50 was used as a treatment condition for analysis of supportive marker. Single culture of each compound was tested in one experiment (duplicates).

### 3.2. Microarray Analysis

Microarray analysis was performed using Human HG-U133A DNA microarrays (*n* = 2/compound; Affymetrix, Santa Clara, CA, USA) and samples treated with MMC, MMS, EMS, CP, ETOP, HU, COLCH, and ADE, in accordance with standard procedure [2]. The Affymetrix Microarray Analysis Suite 5.0 (MAS 5.0, Affymetrix) algorithm was used for calculating the signal value and making a detection call for each probe.

Data from microarray analysis was imported into Spotfire^®^ DecisionSite 9.1.1 for Functional Genomics (Spotfire, Göteborg, Sweden). All signal intensities for each chip were normalized to the average value of all probes on the chip. First, each probe that was judged as presence in at least one microarray among the measured ones was selected for further analysis. The genes whose expression was altered on treatment with the compounds were extracted by calculating the following two statistical parameters for each selected probe: (i) average signal intensity from all microarrays after mean scaling; and (ii) fold change in gene expression between treated and control samples. The following two criteria, namely, signal intensity of >0.1 and fold change of ≥1.5 or ≤0.5, were used to filter probes meeting the two previous statistical criteria.

### 3.3. Analysis of Biological Significance of Genes with Altered Expression on ADE Treatment

The functional annotation online tool DAVID (Database for Annotation, Visualization, and Integrated Discovery, [32]) was used for gene ontology (GO) analysis. A level 5 analysis was adopted for the GO terms of the biological process (threshold count >2). GO terms with *p* values (corrected by Benjamini method) of <0.001 were listed. MetaCore (Thomson Reuters, New York, NY, USA) software was used for functional analysis of genes whose expression was specifically altered on ADE treatment. The network was constructed based on pre-defined molecular interactions in the MetaCore database. A *p* value of <0.01, with the false-discovery rate adjusted using the Benjamini‑Hochberg procedure, was considered statistically significant in the network analysis.

### 3.4. Quantitative RT-PCR Analysis (QPCR)

QPCR was performed on all the samples obtained in the study, as follows. High Capacity cDNA Reverse Transcription Kit (Applied Biosystems, Foster City, CA, USA) was used, according to the manufacturer’s instructions, for the synthesis of cDNA from total RNA; for each reaction, 1 μg of total RNA was used as template in a total volume of 20 μL. For each QPCR, 2 μL of cDNA was used as template, and TaqMan Gene Expression Assay reagents (for *KIF20A*, assay ID: Hs00993573_m1; *GAPDH*, TaqMan Endogenous Controls, predesigned assay reagent, ABI, Applied Biosystems) were used as gene-specific probe and primer sets. TaqMan^®^ Gene Expression Master Mix and ABI PRISM 7900HT System (Applied Biosystems) were used, according to the manufacturer’s instructions, for the reactions and measuring transcript levels, respectively. The conditions for reverse transcription and amplification include 50 °C for 2 min, 95 °C for 10 min, followed by 40 cycles of 95 °C for 15 s and 60 °C for 1 min. The comparative *C*_t_ method was used for calculating the resulting cycle threshold (*C*_t_) values; for each calculation, *GAPDH* was used as an endogenous reference gene for normalizing expression levels of target genes. Values were reported as the means of duplicate analyses.

## 4. Conclusions

In conclusion, *KIF20A* could be used as a biomarker for identifying DNA damage-inducing clastogenicity in conjunction with *CDKN1A*. We have previously reported that *CDKN1A* expression could be used as a biomarker for discriminating chromosomal aberrations that result from DNA damage [2]; it is to be noted, however, that *CDKN1A* expression is affected by the cytotoxic condition. In the present study, *KIF20A* was selected for increasing the accuracy of the discrimination tool, as it was downregulated by all the DNA damage-inducing compounds used in the gene expression analysis. Even though *KIF20A* could be downregulated via *p16* activation independently of *CDKN1A*, we found that *KIF20A* could be effectively used as a supportive biomarker for identifying chromosomal aberrations that result from DNA damage when *CDKN1A* expression is upregulated. We concluded that the utilization of combined assays analyzing *KIF20A* and *CDKN1A* expression is appropriate for discriminating DNA damage-inducing clastogens, because it overcomes their individual drawbacks. A large-scale validation study involving diverse sets of DNA damage-inducing and negative compounds will be needed for studying the association between *CDKN1A* and *KIF20A* in the response to clastogens; in the meantime, the utilization of *KIF20A* biomarker in a simple follow-up assay, in conjunction with *CDKN1A*, can effectively aid appropriate decision making during genotoxicity screening of drug candidates.

## Supplementary Materials

Supplementary materials can be accessed at: http://www.mdpi.com/1422-0067/15/10/17256/s1.

## Supplementary Files

Supplementary File 1
