# Supplementary material for: Combinatorial Measurement of CDKN1A/p21 and KIF20A Expression for Discrimination of DNA Damage-Induced Clastogenicity
Source: Int J Mol Sci. 2014 Sep 26;15(10):17256–69. doi: 10.3390/ijms151017256 (PMC4227160; doi:10.3390/ijms151017256)
Supplement: Supplementary File 1 [file ijms-15-17256-s001.pdf]

Supplementary Information

Table S1. Details of 1236 probes selected from ADE-treated TK6 cells.

Probe Set ID	Gene Title	Gene Symbol	ADE_ave/Corresponding DMSO
200049_at	MYST histone acetyltransferase 2	*MYST2*	0.4269
200050_at	zinc finger protein 146	*ZNF146*	0.3454
200664_s_at	DnaJ (Hsp40) homolog, subfamily B, member 1	*DNAJB1*	0.3713
200666_s_at	DnaJ (Hsp40) homolog, subfamily B, member 1	*DNAJB1*	0.3927
200670_at	X-box binding protein 1	*XBP1*	0.4811
200676_s_at	ubiquitin-conjugating enzyme E2L 3	*UBE2L3*	1.7951
200684_s_at	ubiquitin-conjugating enzyme E2L 3	*UBE2L3*	1.7688
200685_at	splicing factor, arginine/serine-rich 11	*SFRS11*	1.9781
200703_at	dynein, light chain, LC8-type 1	*DYNLL1*	0.4769
200768_s_at	methionine adenosyltransferase II, alpha	*MAT2A*	0.3680
200769_s_at	methionine adenosyltransferase II, alpha	*MAT2A*	0.3544
200798_x_at	myeloid cell leukemia sequence 1 (BCL2-related)	*MCL1*	0.4781
200800_s_at	heat shock 70 kDa protein 1A///heat shock 70 kDa protein 1B	*HSPA1A/HSPA1B*	0.1884
200815_s_at	platelet-activating factor acetylhydrolase, isoform Ib, subunit 1 (45 kDa)	*PAFAH1B1*	0.2655
200868_s_at	ring finger protein 114	*RNF114*	0.4707
200874_s_at	NOP56 ribonucleoprotein homolog (yeast)	*NOP56*	0.4369
200961_at	selenophosphate synthetase 2	*SEPHS2*	0.2579
200992_at	importin 7	*IPO7*	0.4390
200993_at	importin 7	*IPO7*	0.4836
200994_at	importin 7	*IPO7*	0.4067
201041_s_at	dual specificity phosphatase 1	*DUSP1*	0.3398
201155_s_at	mitofusin 2	*MFN2*	0.4877
201196_s_at	adenosylmethionine decarboxylase 1	*AMD1*	0.3825
201197_at	adenosylmethionine decarboxylase 1	*AMD1*	0.3849
201207_at	tumor necrosis factor, alpha-induced protein 1 (endothelial)	*TNFAIP1*	0.3662
201228_s_at	ariadne homolog 2 (Drosophila)	*ARIH2*	1.5010
201243_s_at	ATPase, Na^+/K^+ transporting, beta 1 polypeptide	*ATP1B1*	0.4407
201329_s_at	v-ets erythroblastosis virus E26 oncogene homolog 2 (avian)	*ETS2*	0.3591
201336_at	vesicle-associated membrane protein 3 (cellubrevin)	*VAMP3*	0.4950
201368_at	zinc finger protein 36, C3H type-like 2	*ZFP36L2*	0.2133
201384_s_at	neighbor of BRCA1 gene 1	*NBR1*	0.4572

Table S1. *Cont.*

Probe Set ID	Gene Title	Gene Symbol	ADE_ave/Corresponding DMSO
201394_s_at	RNA binding motif protein 5	*RBM5*	0.4499
201395_at	RNA binding motif protein 5	*RBM5*	0.3968
201417_at	SRY (sex determining region Y)-box 4	*SOX4*	0.2860
201471_s_at	sequestosome 1	*SQSTM1*	0.3927
201473_at	jun B proto-oncogene	*JUNB*	0.3375
201520_s_at	G-rich RNA sequence binding factor 1	*GRSF1*	0.3644
201531_at	zinc finger protein 36, C3H type, homolog (mouse)	*ZFP36*	0.2445
201566_x_at	inhibitor of DNA binding 2, dominant negative helix-loop-helix protein	*ID2*	0.4164
201585_s_at	splicing factor proline/glutamine-rich (polypyrimidine tract binding protein associated)	*SFPQ*	0.3827
201586_s_at	splicing factor proline/glutamine-rich (polypyrimidine tract binding protein associated)	*SFPQ*	0.4774
201602_s_at	protein phosphatase 1, regulatory (inhibitor) subunit 12A	*PPP1R12A*	0.3687
201603_at	protein phosphatase 1, regulatory (inhibitor) subunit 12A	*PPP1R12A*	0.3490
201604_s_at	protein phosphatase 1, regulatory (inhibitor) subunit 12A	*PPP1R12A*	0.3714
201631_s_at	immediate early response 3	*IER3*	0.1905
201644_at	tissue specific transplantation antigen P35B	*TSTA3*	1.5005
201685_s_at	TOX high mobility group box family member 4	*TOX4*	0.4846
201715_s_at	apoptotic chromatin condensation inducer 1	*ACIN1*	0.4405
201734_at	Chloride channel 3	*CLCN3*	0.4467
201735_s_at	chloride channel 3	*CLCN3*	0.4351
201758_at	tumor susceptibility gene 101	*TSG101*	0.4766
201773_at	activity-dependent neuroprotector homeobox	*ADNP*	0.3386
201783_s_at	v-rel reticuloendotheliosis viral oncogene homolog A (avian)	*RELA*	0.2338
201788_at	DEAD (Asp-Glu-Ala-Asp) box polypeptide 42	*DDX42*	0.4994
201823_s_at	ring finger protein 14	*RNF14*	0.3915
201841_s_at	heat shock 27 kDa protein 1	*HSPB1*	1.5951
201850_at	capping protein (actin filament), gelsolin-like	*CAPG*	1.6171
201877_s_at	protein phosphatase 2, regulatory subunit B', gamma isoform	*PPP2R5C*	0.4798

Table S1. *Cont.*

Probe Set ID	Gene Title	Gene Symbol	ADE_ave/Corresponding DMSO
201881_s_at	ariadne homolog, ubiquitin-conjugating enzyme E2 binding protein, 1 (Drosophila)	*ARIH1*	0.4508
201896_s_at	proline/serine-rich coiled-coil 1	*PSRC1*	0.4186
201924_at	AF4/FMR2 family, member 1	*AFF1*	0.3314
201948_at	guanine nucleotide binding protein-like 2 (nucleolar)	*GNL2*	0.4355
201964_at	senataxin	*SETX*	0.4075
201965_s_at	senataxin	*SETX*	0.4586
201987_at	mediator complex subunit 13	*MED13*	0.4943
201992_s_at	kinesin family member 5B	*KIF5B*	0.4833
201993_x_at	heterogeneous nuclear ribonucleoprotein D-like	*HNRPDL*	1.6963
201999_s_at	dynein, light chain, Tctex-type 1	*DYNLT1*	0.4985
202006_at	protein tyrosine phosphatase, non-receptor type 12	*PTPN12*	0.3970
202033_s_at	RB1-inducible coiled-coil 1	*RB1CC1*	0.4034
202034_x_at	RB1-inducible coiled-coil 1	*RB1CC1*	0.4239
202049_s_at	zinc finger, MYM-type 4	*ZMYM4*	0.3908
202066_at	protein tyrosine phosphatase, receptor type, f polypeptide (PTPRF), interacting protein (liprin), alpha 1	*PPFIA1*	0.3380
202081_at	immediate early response 2	*IER2*	0.2585
202094_at	baculoviral IAP repeat-containing 5	*BIRC5*	1.7418
202096_s_at	translocator protein (18 kDa)	*TSPO*	1.5257
202097_at	nucleoporin 153 kDa	*NUP153*	0.4282
202113_s_at	sorting nexin 2	*SNX2*	0.4194
202114_at	sorting nexin 2	*SNX2*	0.3759
202116_at	D4, zinc and double PHD fingers family 2	*DPF2*	0.2467
202124_s_at	trafficking protein, kinesin binding 2	*TRAK2*	0.4927
202136_at	zinc finger, MYND domain containing 11	*ZMYND11*	0.4497
202153_s_at	nucleoporin 62 kDa	*NUP62*	0.3135
202155_s_at	nucleoporin 214 kDa	*NUP214*	0.4488
202159_at	phenylalanyl-tRNA synthetase, alpha subunit	*FARSA*	0.4497
202160_at	CREB binding protein	*CREBBP*	0.3249
202176_at	excision repair cross-complementing rodent repair deficiency, complementation group 3 (xeroderma pigmentosum group B complementing)	*ERCC3*	0.3918
202190_at	cleavage stimulation factor, 3' pre-RNA, subunit 1, 50 kDa	*CSTF1*	0.3357

Table S1. *Cont.*

Probe Set ID	Gene Title	Gene Symbol	ADE_ave/Corresponding DMSO
202215_s_at	nuclear transcription factor Y, gamma	*NFYC*	0.4856
202218_s_at	fatty acid desaturase 2	*FADS2*	1.5531
202226_s_at	v-crk sarcoma virus CT10 oncogene homolog (avian)	*CRK*	0.4647
202241_at	tribbles homolog 1 (Drosophila)	*TRIB1*	0.2160
202258_s_at	NEDD4 binding protein 2-like 2	*N4BP2L2*	0.4758
202260_s_at	syntaxin binding protein 1	*STXBP1*	0.4521
202266_at	TRAF and TNF receptor associated protein	*TTRAP*	0.3915
202272_s_at	F-box protein 28	*FBXO28*	0.2530
202293_at	stromal antigen 1	*STAG1*	0.4698
202301_s_at	arginine/serine-rich coiled-coil 2	*RSRC2*	1.7114
202302_s_at	arginine/serine-rich coiled-coil 2	*RSRC2*	1.7024
202333_s_at	ubiquitin-conjugating enzyme E2B (RAD6 homolog)	*UBE2B*	0.4683
202348_s_at	torsin family 1, member A (torsin A)	*TOR1A*	0.3966
202349_at	torsin family 1, member A (torsin A)	*TOR1A*	0.4713
202351_at	integrin, alpha V (vitronectin receptor, alpha polypeptide, antigen CD51)	*ITGAV*	0.4170
202375_at	SEC24 family, member D (S. cerevisiae)	*SEC24D*	0.4291
202393_s_at	Kruppel-like factor 10	*KLF10*	3.2931
202417_at	kelch-like ECH-associated protein 1	*KEAP1*	0.4212
202431_s_at	v-myc myelocytomatosis viral oncogene homolog (avian)	*MYC*	0.1103
202495_at	tubulin folding cofactor C	*TBCC*	0.4629
202506_at	sperm specific antigen 2	*SSFA2*	0.4723
202535_at	Fas (TNFRSF6)-associated via death domain	*FADD*	0.1025
202547_s_at	Rho guanine nucleotide exchange factor (GEF) 7	*ARHGEF7*	0.3125
202548_s_at	Rho guanine nucleotide exchange factor (GEF) 7	*ARHGEF7*	0.4518
202553_s_at	SYF2 homolog, RNA splicing factor (S. cerevisiae)	*SYF2*	1.5262
202581_at	heat shock 70 kDa protein 1A/heat shock 70 kDa protein 1B	*HSPA1A/HSPA1B*	0.2554
202582_s_at	RAN binding protein 9	*RANBP9*	0.3393
202583_s_at	RAN binding protein 9	*RANBP9*	0.3233
202599_s_at	nuclear receptor interacting protein 1	*NRIP1*	0.4146
202613_at	CTP synthase	*CTPS*	0.4702
202615_at	Guanine nucleotide binding protein (G protein), q polypeptide	*GNAQ*	1.7111

Table S1. *Cont.*

Probe Set ID	Gene Title	Gene Symbol	ADE_ave/Corresponding DMSO
202629_at	amyloid beta precursor protein (cytoplasmic tail) binding protein 2	*APPBP2*	0.3407
202637_s_at	intercellular adhesion molecule 1	*ICAM1*	0.4681
202638_s_at	intercellular adhesion molecule 1	*ICAM1*	0.4104
202640_s_at	RAN binding protein 3	*RANBP3*	0.4790
202644_s_at	tumor necrosis factor, alpha-induced protein 3	*TNFAIP3*	0.4757
202648_at	–	–	0.4621
202653_s_at	membrane-associated ring finger (C3HC4) 7	*MARCH7*	0.4225
202654_x_at	membrane-associated ring finger (C3HC4) 7	*MARCH7*	0.4290
202679_at	Niemann-Pick disease, type C1	*NPC1*	0.3191
202689_at	RNA binding motif protein 15B	*RBM15B*	0.3662
202693_s_at	serine/threonine kinase 17a	*STK17A*	0.4783
202702_at	tripartite motif-containing 26	*TRIM26*	0.2855
202703_at	dual specificity phosphatase 11 (RNA/RNP complex 1-interacting)	*DUSP11*	0.3392
202704_at	transducer of ERBB2, 1	*TOB1*	0.3794
202706_s_at	uridine monophosphate synthetase	*UMPS*	0.3140
202717_s_at	cell division cycle 16 homolog (S. cerevisiae)	*CDC16*	0.4932
202750_s_at	tuftelin interacting protein 11	*TFIP11*	0.4394
202769_at	cyclin G2	*CCNG2*	1.9691
202776_at	deoxynucleotidyltransferase, terminal, interacting protein 2	*DNTTIP2*	0.4339
202777_at	soc-2 suppressor of clear homolog (C. elegans)	*SHOC2*	0.4641
202798_at	SEC24 family, member B (S. cerevisiae)	*SEC24B*	0.3143
202814_s_at	hexamethylene bis-acetamide inducible 1	*HEXIM1*	0.1837
202816_s_at	synovial sarcoma translocation, chromosome 18	*SS18*	0.4143
202817_s_at	synovial sarcoma translocation, chromosome 18	*SS18*	0.4078
202832_at	GRIP and coiled-coil domain containing 2	*GCC2*	0.4888
202841_x_at	opioid growth factor receptor	*OGFR*	0.4351
202866_at	DnaJ (Hsp40) homolog, subfamily B, member 12	*DNAJB12*	0.4760
202871_at	TNF receptor-associated factor 4	*TRAF4*	0.4461
202872_at	ATPase, H^+ transporting, lysosomal 42 kDa, V1 subunit C1	*ATP6V1C1*	0.4570
202887_s_at	DNA-damage-inducible transcript 4	*DDIT4*	0.3705
202915_s_at	family with sequence similarity 20, member B	*FAM20B*	0.4922
202916_s_at	family with sequence similarity 20, member B	*FAM20B*	0.4940
202918_s_at	MOB1, Mps One Binder kinase activator-like 3 (yeast)	*MOBKL3*	0.2046

Table S1. *Cont.*

Probe Set ID	Gene Title	Gene Symbol	ADE_ave/Corresponding DMSO
202919_at	MOB1, Mps One Binder kinase activator-like 3 (yeast)	*MOBKL3*	0.4753
202922_at	glutamate-cysteine ligase, catalytic subunit	*GCLC*	0.4884
202923_s_at	glutamate-cysteine ligase, catalytic subunit	*GCLC*	0.3841
202947_s_at	glycophorin C (Gerbich blood group)	*GYPC*	1.5039
202949_s_at	four and a half LIM domains 2	*FHL2*	0.3901
202951_at	serine/threonine kinase 38	*STK38*	0.3901
202955_s_at	ADP-ribosylation factor guanine nucleotide-exchange factor 1 (brefeldin A-inhibited)	*ARFGEF1*	0.4811
202956_at	ADP-ribosylation factor guanine nucleotide-exchange factor 1 (brefeldin A-inhibited)	*ARFGEF1*	0.4340
202968_s_at	dual-specificity tyrosine-(Y)-phosphorylation regulated kinase 2	*DYRK2*	0.3474
202969_at	dual-specificity tyrosine-(Y)-phosphorylation regulated kinase 2	*DYRK2*	0.3440
202979_s_at	CREB/ATF bZIP transcription factor	*CREBZF*	1.5614
202984_s_at	BCL2-associated athanogene 5	*BAG5*	0.4437
202985_s_at	BCL2-associated athanogene 5	*BAG5*	0.3705
203026_at	zinc finger and BTB domain containing 5	*ZBTB5*	0.4976
203043_at	zinc finger, BED-type containing 1	*ZBED1*	0.3960
203044_at	chondroitin sulfate synthase 1	*CHSY1*	0.3372
203051_at	bromo adjacent homology domain containing 1	*BAHD1*	0.4627
203053_at	breast carcinoma amplified sequence 2	*BCAS2*	0.3350
203063_at	protein phosphatase 1F (PP2C domain containing)	*PPM1F*	1.5867
203064_s_at	forkhead box K2	*FOXK2*	0.4265
203065_s_at	caveolin 1, caveolae protein, 22 kDa	*CAV1*	1.5817
203073_at	component of oligomeric golgi complex 2	*COG2*	0.4563
203077_s_at	SMAD family member 2	*SMAD2*	0.4545
203087_s_at	kinesin heavy chain member 2A	*KIF2A*	0.4665
203102_s_at	mannosyl (alpha-1,6-)-glycoprotein beta-1,2-*N*-acetylglucosaminyltransferase	*MGAT2*	0.1396
203112_s_at	Wolf-Hirschhorn syndrome candidate 2	*WHSC2*	0.4796
203135_at	TATA box binding protein	*TBP*	0.2283
203137_at	Wilms tumor 1 associated protein	*WTAP*	0.4961
203156_at	A kinase (PRKA) anchor protein 11	*AKAP11*	0.3887
203164_at	solute carrier family 33 (acetyl-CoA transporter), member 1	*SLC33A1*	0.3449

Table S1. *Cont.*

Probe Set ID	Gene Title	Gene Symbol	ADE_ave/Corresponding DMSO
203165_s_at	solute carrier family 33 (acetyl-CoA transporter), member 1	*SLC33A1*	0.3493
203182_s_at	SFRS protein kinase 2	*SRPK2*	0.3499
203194_s_at	nucleoporin 98 kDa	*NUP98*	0.4604
203195_s_at	nucleoporin 98 kDa	*NUP98*	0.3578
203205_at	lysine (K)-specific demethylase 4A	*KDM4A*	0.3473
203214_x_at	cell division cycle 2, G1 to S and G2 to M	*CDC2*	0.4866
203231_s_at	ataxin 1	*ATXN1*	2.3701
203232_s_at	ataxin 1	*ATXN1*	2.0669
203239_s_at	CCR4-NOT transcription complex, subunit 3	*CNOT3*	0.4708
203250_at	RNA binding motif protein 16	*RBM16*	0.3761
203265_s_at	mitogen-activated protein kinase 4	*MAP2K4*	0.4888
203266_s_at	mitogen-activated protein kinase 4	*MAP2K4*	0.4330
203274_at	coagulation factor VIII-associated (intronic transcript) 1/coagulation factor VIII-associated (intronic transcript) 2/coagulation factor VIII-associated (intronic transcript) 3	*F8A1/F8A2/F8A3*	0.2433
203291_at	CCR4-NOT transcription complex, subunit 4	*CNOT4*	0.3175
203293_s_at	lectin, mannose-binding, 1	*LMAN1*	1.6687
203310_at	syntaxin binding protein 3	*STXBP3*	0.4179
203311_s_at	ADP-ribosylation factor 6	*ARF6*	0.4786
203317_at	pleckstrin and Sec7 domain containing 4	*PSD4*	0.4269
203318_s_at	zinc finger protein 148	*ZNF148*	0.4724
203319_s_at	zinc finger protein 148	*ZNF148*	0.4333
203341_at	CCAAT/enhancer binding protein (C/EBP), zeta	*CEBPZ*	0.1951
203345_s_at	metal response element binding transcription factor 2	*MTF2*	0.4779
203347_s_at	metal response element binding transcription factor 2	*MTF2*	0.4256
203351_s_at	origin recognition complex, subunit 4-like (yeast)	*ORC4L*	0.3239
203356_at	calpain 7	*CAPN7*	0.3266
203373_at	suppressor of cytokine signaling 2	*SOCS2*	0.3872
203377_s_at	cell division cycle 40 homolog (S. cerevisiae)	*CDC40*	0.3825
203391_at	FK506 binding protein 2, 13 kDa	*FKBP2*	1.5554
203403_s_at	ring finger protein (C3H2C3 type) 6	*RNF6*	0.4043
203420_at	family with sequence similarity 8, member A1	*FAM8A1*	0.3459
203427_at	ASF1 anti-silencing function 1 homolog A (S. cerevisiae)	*ASF1A*	0.3697
203428_s_at	ASF1 anti-silencing function 1 homolog A (S. cerevisiae)	*ASF1A*	0.2908

Table S1. *Cont.*

Probe Set ID	Gene Title	Gene Symbol	ADE_ave/Corresponding DMSO
203429_s_at	chromosome 1 open reading frame 9	*C1orf9*	0.3497
203447_at	proteasome (prosome, macropain) 26S subunit, non-ATPase, 5	*PSMD5*	0.4588
203455_s_at	spermidine/spermine *N*1-acetyltransferase 1	*SAT1*	1.5004
203481_at	family with sequence similarity 178, member A	*FAM178A*	0.4434
203486_s_at	armadillo repeat containing 8	*ARMC8*	0.3010
203487_s_at	armadillo repeat containing 8	*ARMC8*	0.3237
203496_s_at	mediator complex subunit 1	*MED1*	0.4900
203521_s_at	zinc finger protein 318	*ZNF318*	0.2566
203531_at	cullin 5	*CUL5*	0.4862
203544_s_at	signal transducing adaptor molecule (SH3 domain and ITAM motif) 1	*STAM*	0.3966
203552_at	mitogen-activated protein kinase 5	*MAP4K5*	0.4153
203553_s_at	mitogen-activated protein kinase 5	*MAP4K5*	0.3291
203556_at	zinc fingers and homeoboxes 2	*ZHX2*	0.3502
203565_s_at	menage a trois homolog 1, cyclin H assembly factor (Xenopus laevis)	*MNAT1*	0.4492
203567_s_at	tripartite motif-containing 38	*TRIM38*	0.4804
203569_s_at	oral-facial-digital syndrome 1	*OFD1*	0.4891
203583_at	unc-50 homolog (C. elegans)	*UNC50*	0.3815
203584_at	tetratricopeptide repeat domain 35	*TTC35*	0.3693
203597_s_at	WW domain binding protein 4 (formin binding protein 21)	*WBP4*	0.3340
203598_s_at	WW domain binding protein 4 (formin binding protein 21)	*WBP4*	0.3515
203599_s_at	WW domain binding protein 4 (formin binding protein 21)	*WBP4*	0.3025
203600_s_at	chromosome 4 open reading frame 8	*C4orf8*	0.4198
203611_at	telomeric repeat binding factor 2	*TERF2*	0.3710
203612_at	bystin-like	*BYSL*	0.4392
203614_at	UTP14, U3 small nucleolar ribonucleoprotein, homolog C (yeast)	*UTP14C*	0.3345
203622_s_at	partner of NOB1 homolog (S. cerevisiae)	*PNO1*	0.4730
203635_at	Down syndrome critical region gene 3	*DSCR3*	0.4796
203659_s_at	tripartite motif-containing 13	*TRIM13*	0.2809
203660_s_at	pericentrin	*PCNT*	0.4972
203665_at	heme oxygenase (decycling) 1	*HMOX1*	0.4421
203688_at	polycystic kidney disease 2 (autosomal dominant)	*PKD2*	0.4598

Table S1. *Cont.*

Probe Set ID	Gene Title	Gene Symbol	ADE_ave/Corresponding DMSO
203690_at	tubulin, gamma complex associated protein 3	*TUBGCP3*	0.2094
203694_s_at	DEAH (Asp-Glu-Ala-His) box polypeptide 16	*DHX16*	0.4642
203732_at	thyroid hormone receptor interactor 4	*TRIP4*	0.3633
203734_at	forkhead box J2	*FOXJ2*	0.4950
203737_s_at	peroxisome proliferator-activated receptor γ, coactivator-related 1	*PPRC1*	0.3377
203739_at	zinc finger protein 217	*ZNF217*	0.1756
203751_x_at	jun D proto-oncogene	*JUND*	0.4293
203752_s_at	jun D proto-oncogene	*JUND*	0.3675
203755_at	budding uninhibited by benzimidazoles 1 homolog beta (yeast)	*BUB1B*	0.4048
203769_s_at	steroid sulfatase (microsomal), isozyme S	*STS*	1.9268
203828_s_at	interleukin 32	*IL32*	1.5638
203829_at	elongation protein 4 homolog (S. cerevisiae)	*ELP4*	0.4226
203836_s_at	mitogen-activated protein kinase 5	*MAP3K5*	0.4018
203837_at	mitogen-activated protein kinase 5	*MAP3K5*	0.4427
203846_at	tripartite motif-containing 32	*TRIM32*	0.2318
203881_s_at	dystrophin	*DMD*	1.8377
203893_at	TAF9 RNA polymerase II, TATA box binding protein (TBP)-associated factor, 32 kDa	*TAF9*	0.3663
203909_at	solute carrier family 9 (sodium/hydrogen exchanger), member 6	*SLC9A6*	0.4960
203916_at	*N*-deacetylase/*N*-sulfotransferase (heparan glucosaminyl) 2	*NDST2*	0.2485
203925_at	glutamate-cysteine ligase, modifier subunit	*GCLM*	0.3640
203927_at	nuclear factor of kappa light polypeptide gene enhancer in B-cells inhibitor, epsilon	*NFKBIE*	0.3363
203944_x_at	butyrophilin, subfamily 2, member A1	*BTN2A1*	0.3412
203947_at	cleavage stimulation factor, 3' pre-RNA, subunit 3, 77 kDa	*CSTF3*	0.2995
203966_s_at	protein phosphatase 1A (formerly 2C), magnesium-dependent, alpha isoform	*PPM1A*	0.4284
203967_at	cell division cycle 6 homolog (S. cerevisiae)	*CDC6*	0.2992
203968_s_at	cell division cycle 6 homolog (S. cerevisiae)	*CDC6*	0.3440
203975_s_at	chromatin assembly factor 1, subunit A (p150)	*CHAF1A*	0.3548
203976_s_at	chromatin assembly factor 1, subunit A (p150)	*CHAF1A*	0.1815
203978_at	nucleotide binding protein 1 (MinD homolog, E. coli)	*NUBP1*	0.4403
203984_s_at	caspase 9, apoptosis-related cysteine peptidase	*CASP9*	0.3815
204003_s_at	nucleoporin like 2	*NUPL2*	0.3678

Table S1. *Cont.*

Probe Set ID	Gene Title	Gene Symbol	ADE_ave/Corresponding DMSO
204011_at	sprouty homolog 2 (Drosophila)	*SPRY2*	0.4025
204023_at	replication factor C (activator 1) 4, 37 kDa	*RFC4*	0.3455
204033_at	thyroid hormone receptor interactor 13	*TRIP13*	0.3302
204065_at	carbohydrate sulfotransferase 10	*CHST10*	0.3135
204067_at	sulfite oxidase	*SUOX*	0.4240
204080_at	target of EGR1, member 1 (nuclear)	*TOE1*	0.3750
204089_x_at	mitogen-activated protein kinase 4	*MAP3K4*	0.2911
204092_s_at	aurora kinase A	*AURKA*	0.2974
204094_s_at	TSC22 domain family, member 2	*TSC22D2*	0.1922
204118_at	CD48 molecule	*CD48*	1.5169
204159_at	cyclin-dependent kinase inhibitor 2C (p18, inhibits CDK4)	*CDKN2C*	0.3561
204162_at	NDC80 homolog, kinetochore complex component (S. cerevisiae)	*NDC80*	0.2141
204173_at	myosin, light chain 6B, alkali, smooth muscle and non-muscle	*MYL6B*	1.6219
204175_at	zinc finger protein 593	*ZNF593*	0.3686
204194_at	BTB and CNC homology 1, basic leucine zipper transcription factor 1	*BACH1*	2.0975
204197_s_at	runt-related transcription factor 3	*RUNX3*	0.2983
204198_s_at	runt-related transcription factor 3	*RUNX3*	0.3713
204203_at	CCAAT/enhancer binding protein (C/EBP), γ	*CEBPG*	0.3157
204216_s_at	zinc finger CCCH-type containing 14	*ZC3H14*	0.3409
204224_s_at	GTP cyclohydrolase 1	*GCH1*	0.4439
204233_s_at	choline kinase alpha	*CHKA*	0.4624
204244_s_at	DBF4 homolog (S. cerevisiae)	*DBF4*	0.4184
204245_s_at	ribonuclease P/MRP 14 kDa subunit	*RPP14*	0.3637
204258_at	chromodomain helicase DNA binding protein 1	*CHD1*	0.4922
204263_s_at	carnitine palmitoyltransferase 2	*CPT2*	0.4473
204264_at	carnitine palmitoyltransferase 2	*CPT2*	0.4480
204265_s_at	G-protein signaling modulator 3 (AGS3-like, C. elegans)	*GPSM3*	1.7744
204266_s_at	choline kinase alpha	*CHKA*	0.4463
204291_at	zinc finger protein 518A	*ZNF518A*	0.3049
204317_at	G-2 and S-phase expressed 1	*GTSE1*	0.4979
204318_s_at	G-2 and S-phase expressed 1	*GTSE1*	0.4962
204335_at	coiled-coil domain containing 94	*CCDC94*	0.3634
204336_s_at	regulator of G-protein signaling 19	*RGS19*	0.4863
204350_s_at	mediator complex subunit 7	*MED7*	0.2429
204355_at	DEAH (Asp-Glu-Ala-His) box polypeptide 30	*DHX30*	0.4984

Table S1. *Cont.*

Probe Set ID	Gene Title	Gene Symbol	ADE_ave/Corresponding DMSO
204383_at	DiGeorge syndrome critical region gene 14	*DGCR14*	0.4738
204391_x_at	tripartite motif-containing 24	*TRIM24*	0.3053
204396_s_at	G protein-coupled receptor kinase 5	*GRK5*	0.4897
204407_at	transcription termination factor, RNA polymerase II	*TTF2*	0.4629
204434_at	spermatogenesis associated 2	*SPATA2*	1.5021
204435_at	nucleoporin like 1	*NUPL1*	1.7656
204474_at	zinc finger protein 142	*ZNF142*	0.4552
204478_s_at	RAB interacting factor	*RABIF*	0.4396
204489_s_at	CD44 molecule (Indian blood group)	*CD44*	1.5509
204494_s_at	chromosome 15 open reading frame 39	*C15orf39*	0.2663
204495_s_at	chromosome 15 open reading frame 39	*C15orf39*	0.2546
204496_at	striatin, calmodulin binding protein 3	*STRN3*	0.3717
204507_s_at	protein phosphatase 3 (formerly 2B), regulatory subunit B, alpha isoform	*PPP3R1*	0.4845
204516_at	ataxin 7	*ATXN7*	0.4740
204523_at	zinc finger protein 140	*ZNF140*	0.4399
204544_at	Hermansky-Pudlak syndrome 5	*HPS5*	0.3939
204562_at	interferon regulatory factor 4	*IRF4*	0.4713
204563_at	selectin L	*SELL*	1.7303
204605_at	cell growth regulator with ring finger domain 1	*CGRRF1*	0.3236
204618_s_at	GA binding protein transcription factor, β subunit 1	*GABPB1*	0.4782
204630_s_at	golgi SNAP receptor complex member 1	*GOSR1*	0.4274
204674_at	lymphoid-restricted membrane protein	*LRMP*	1.5645
204676_at	transmembrane protein 186	*TMEM186*	0.2083
204689_at	hematopoietically expressed homeobox	*HHEX*	0.3754
204695_at	cell division cycle 25 homolog A (S. pombe)	*CDC25A*	0.2856
204699_s_at	chromosome 1 open reading frame 107	*C1orf107*	0.3324
204700_x_at	chromosome 1 open reading frame 107	*C1orf107*	0.3693
204702_s_at	nuclear factor (erythroid-derived 2)-like 3	*NFE2L3*	0.4741
204709_s_at	kinesin family member 23	*KIF23*	0.4891
204715_at	pannexin 1	*PANX1*	0.3630
204747_at	interferon-induced protein with tetratricopeptide repeats 3	*IFIT3*	0.4178
204775_at	chromatin assembly factor 1, subunit B (p60)	*CHAF1B*	0.4739
204794_at	dual specificity phosphatase 2	*DUSP2*	0.3054
204799_at	zinc finger, BED-type containing 4	*ZBED4*	0.3342
204826_at	cyclin F	*CCNF*	0.1492
204832_s_at	bone morphogenetic protein receptor, type IA	*BMPR1A*	0.4660

Table S1. *Cont.*

Probe Set ID	Gene Title	Gene Symbol	ADE_ave/Corresponding DMSO
204853_at	origin recognition complex, subunit 2-like (yeast)	*ORC2L*	0.4717
204859_s_at	apoptotic peptidase activating factor 1	*APAF1*	0.4443
204868_at	immature colon carcinoma transcript 1	*ICT1*	0.3463
204899_s_at	Sin3A-associated protein, 30 kDa	*SAP30*	0.3918
204900_x_at	Sin3A-associated protein, 30 kDa	*SAP30*	0.3936
204928_s_at	solute carrier family 10 (sodium/bile acid cotransporter family), member 3	*SLC10A3*	0.3722
204962_s_at	centromere protein A	*CENPA*	0.4072
204995_at	cyclin-dependent kinase 5, regulatory subunit 1 (p35)	*CDK5R1*	0.4531
205027_s_at	mitogen-activated protein kinase 8	*MAP3K8*	2.2556
205034_at	cyclin E2	*CCNE2*	0.3293
205042_at	glucosamine UDP-*N*-acetyl)-2-epimerase/ *N*-acetylmannosamine kinase	*GNE*	0.3959
205046_at	centromere protein E, 312 kDa	*CENPE*	0.3278
205063_at	survival of motor neuron protein interacting protein 1	*SIP1*	0.2996
205071_x_at	X-ray repair complementing defective repair in Chinese hamster cells 4	*XRCC4*	0.4442
205089_at	zinc finger protein 7	*ZNF7*	0.3153
205097_at	solute carrier family 26 (sulfate transporter), member 2	*SLC26A2*	0.4198
205114_s_at	chemokine (C-C motif) ligand 3/chemokine (C-C motif) ligand 3-like 1 /chemokine (C-C motif) ligand 3-like 3	*CCL3/CCL3L1/ CCL3L3*	0.0650
205126_at	vaccinia related kinase 2	*VRK2*	0.4551
205134_s_at	nuclear fragile X mental retardation protein interacting protein 1	*NUFIP1*	0.2150
205135_s_at	nuclear fragile X mental retardation protein interacting protein 1	*NUFIP1*	0.3208
205136_s_at	nuclear fragile X mental retardation protein interacting protein 1	*NUFIP1*	0.4554
205153_s_at	CD40 molecule, TNF receptor superfamily member 5	*CD40*	0.4922
205173_x_at	CD58 molecule	*CD58*	0.4356
205178_s_at	retinoblastoma binding protein 6	*RBBP6*	0.2679
205191_at	retinitis pigmentosa 2 (X-linked recessive)	*RP2*	0.4516
205218_at	polymerase (RNA) III (DNA directed) polypeptide F, 39 kDa	*POLR3F*	0.3990

Table S1. *Cont.*

Probe Set ID	Gene Title	Gene Symbol	ADE_ave/Corresponding DMSO
205231_s_at	epilepsy, progressive myoclonus type 2A, Lafora disease (laforin)	*EPM2A*	0.4948
205235_s_at	kinesin family member 20B	*KIF20B*	0.3276
205241_at	SCO cytochrome oxidase deficient homolog 2 (yeast)	*SCO2*	0.2972
205251_at	period homolog 2 (Drosophila)	*PER2*	0.3907
205264_at	CD3e molecule, epsilon associated protein	*CD3EAP*	0.3621
205296_at	retinoblastoma-like 1 (p107)	*RBL1*	0.3843
205335_s_at	signal recognition particle 19 kDa	*SRP19*	0.3209
205340_at	zinc finger and BTB domain containing 24	*ZBTB24*	0.3528
205345_at	BRCA1 associated RING domain 1	*BARD1*	0.4616
205419_at	G protein-coupled receptor 183	*GPR183*	0.1704
205429_s_at	membrane protein, palmitoylated 6 (MAGUK p55 subfamily member 6)	*MPP6*	0.3866
205443_at	small nuclear RNA activating complex, polypeptide 1, 43 kDa	*SNAPC1*	0.2233
205519_at	WD repeat domain 76	*WDR76*	0.2430
205526_s_at	katanin p60 (ATPase-containing) subunit A 1	*KATNA1*	0.3454
205527_s_at	gem (nuclear organelle) associated protein 4	*GEMIN4*	0.0927
205548_s_at	BTG family, member 3	*BTG3*	0.4853
205562_at	ribonuclease P/MRP 38 kDa subunit	*RPP38*	0.4032
205596_s_at	SMAD specific E3 ubiquitin protein ligase 2	*SMURF2*	0.4844
205599_at	TNF receptor-associated factor 1	*TRAF1*	0.2434
205621_at	alkB, alkylation repair homolog 1 (E. coli)	*ALKBH1*	0.4517
205633_s_at	aminolevulinate, delta-, synthase 1	*ALAS1*	0.2504
205661_s_at	FAD1 flavin adenine dinucleotide synthetase homolog (S. cerevisiae)	*FLAD1*	0.4569
205664_at	KIN, antigenic determinant of recA protein homolog (mouse)	*KIN*	0.2606
205667_at	Werner syndrome, RecQ helicase-like	*WRN*	0.4692
205707_at	interleukin 17 receptor A	*IL17RA*	0.4173
205733_at	Bloom syndrome, RecQ helicase-like	*BLM*	0.3418
205739_x_at	zinc finger protein 107	*ZNF107*	0.2381
205748_s_at	ring finger protein 126	*RNF126*	0.4356
205750_at	biphenyl hydrolase-like (serine hydrolase)	*BPHL*	1.5994
205811_at	polymerase (DNA directed), gamma 2, accessory subunit	*POLG2*	0.3510
205854_at	tubby like protein 3	*TULP3*	0.3663
205875_s_at	three prime repair exonuclease 1	*TREX1*	0.3701

Table S1. *Cont.*

Probe Set ID	Gene Title	Gene Symbol	ADE_ave/Corresponding DMSO
205909_at	polymerase (DNA directed), epsilon 2 (p59 subunit)	*POLE2*	0.4623
205928_at	zinc finger protein 443	*ZNF443*	0.4204
205930_at	general transcription factor IIE, polypeptide 1, alpha 56 kDa	*GTF2E1*	0.4764
205981_s_at	inhibitor of growth family, member 2	*ING2*	0.4386
206003_at	centrosomal protein 135 kDa	*CEP135*	0.4687
206015_s_at	forkhead box J3	*FOXJ3*	0.4180
206052_s_at	stem-loop binding protein	*SLBP*	0.4968
206096_at	zinc finger protein 35	*ZNF35*	0.4270
206140_at	LIM homeobox 2	*LHX2*	0.4788
206173_x_at	GA binding protein transcription factor, beta subunit 1	*GABPB1*	0.3343
206219_s_at	vav 1 guanine nucleotide exchange factor	*VAV1*	0.4337
206245_s_at	influenza virus NS1A binding protein	*IVNS1ABP*	1.5584
206337_at	chemokine (C-C motif) receptor 7	*CCR7*	0.3956
206364_at	kinesin family member 14	*KIF14*	0.3452
206451_at	TBCC domain containing 1	*TBCCD1*	0.3240
206468_s_at	methyltransferase like 13	*METTL13*	0.3090
206472_s_at	transducin-like enhancer of split 3 (E(sp1) homolog, Drosophila)	*TLE3*	0.4974
206715_at	transcription factor EC	*TFEC*	2.8486
206723_s_at	lysophosphatidic acid receptor 2	*LPAR2*	1.7424
206809_s_at	heterogeneous nuclear ribonucleoprotein A3/ heterogeneous nuclear ribonucleoprotein A3 pseudogene 1	*HNRNPA3/HNRNP A3P1*	1.8673
206845_s_at	ring finger protein 40	*RNF40*	0.4356
206860_s_at	missing oocyte, meiosis regulator, homolog (Drosophila)	*MIOS*	0.3871
206875_s_at	STE20-like kinase (yeast)	*SLK*	0.3724
206928_at	zinc finger protein 124	*ZNF124*	0.3201
206934_at	signal-regulatory protein beta 1	*SIRPB1*	2.0646
207000_s_at	protein phosphatase 3 (formerly 2B), catalytic subunit, gamma isoform	*PPP3CC*	0.4052
207098_s_at	mitofusin 1	*MFN1*	0.3193
207131_x_at	gamma-glutamyltransferase 1	*GGT1*	1.5684
207153_s_at	glomulin, FKBP associated protein	*GLMN*	0.4633
207318_s_at	cell division cycle 2-like 5 (cholinesterase-related cell division controller)	*CDC2L5*	0.2190
207338_s_at	zinc finger protein 200	*ZNF200*	0.2695

Table S1. *Cont.*

Probe Set ID	Gene Title	Gene Symbol	ADE_ave/Corresponding DMSO
207390_s_at	smoothelin	*SMTN*	0.3577
207391_s_at	phosphatidylinositol-4-phosphate 5-kinase, type I, alpha	*PIP5K1A*	0.3147
207405_s_at	RAD17 homolog (S. pombe)	*RAD17*	0.4737
207480_s_at	Meis homeobox 2	*MEIS2*	0.4632
207513_s_at	zinc finger protein 189	*ZNF189*	0.4484
207564_x_at	O-linked *N*-acetylglucosamine (GlcNAc) transferase	*OGT*	1.5844
207574_s_at	growth arrest and DNA-damage-inducible, beta	*GADD45B*	1.6085
207614_s_at	cullin 1	*CUL1*	0.3848
207719_x_at	centrosomal protein 170 kDa	*CEP170*	0.4877
207730_x_at	–	–	1.5112
207738_s_at	NCK-associated protein 1	*NCKAP1*	0.2958
207740_s_at	nucleoporin 62 kDa	*NUP62*	0.4599
207826_s_at	inhibitor of DNA binding 3, dominant negative helix-loop-helix protein	*ID3*	0.3906
207830_s_at	protein phosphatase 1, regulatory (inhibitor) subunit 8	*PPP1R8*	0.3388
207842_s_at	cancer susceptibility candidate 3	*CASC3*	0.4487
207845_s_at	anaphase promoting complex subunit 10	*ANAPC10*	0.4598
207861_at	chemokine (C-C motif) ligand 22	*CCL22*	1.5772
207877_s_at	nuclear VCP-like	*NVL*	0.4605
207922_s_at	macrophage erythroblast attacher	*MAEA*	0.4022
207945_s_at	casein kinase 1, delta	*CSNK1D*	0.4897
207980_s_at	Cbp/p300-interacting transactivator, with Glu/Asp-rich carboxy-terminal domain, 2	*CITED2*	0.4785
208042_at	angiogenic factor with G patch and FHA domains 1	*AGGF1*	0.3896
208079_s_at	aurora kinase A	*AURKA*	0.2976
208089_s_at	tudor domain containing 3	*TDRD3*	0.4068
208238_x_at	–	–	1.9098
208284_x_at	gamma-glutamyltransferase 1	*GGT1*	1.5608
208290_s_at	eukaryotic translation initiation factor 5	*EIF5*	0.4803
208309_s_at	mucosa associated lymphoid tissue lymphoma translocation gene 1	*MALT1*	0.4632
208398_s_at	TBP-like 1	*TBPL1*	0.4336
208406_s_at	GRB2-related adaptor protein 2	*GRAP2*	0.4985
208523_x_at	histone cluster 1, H2bi	*HIST1H2BI*	1.8311
208687_x_at	heat shock 70 kDa protein 8	*HSPA8*	0.4837
208708_x_at	eukaryotic translation initiation factor 5	*EIF5*	0.4742

Table S1. *Cont.*

Probe Set ID	Gene Title	Gene Symbol	ADE_ave/Corresponding DMSO
208725_at	eukaryotic translation initiation factor 2, subunit 2 beta, 38 kDa	*EIF2S2*	1.8373
208763_s_at	TSC22 domain family, member 3	*TSC22D3*	1.6888
208773_s_at	ankyrin repeat and KH domain containing 1 /ANKHD1-EIF4EBP3 readthrough transcript	*ANKHD1/ANKHD1 -EIF4EBP3*	0.4969
208878_s_at	p21 protein (Cdc42/Rac)-activated kinase 2	*PAK2*	0.3237
208903_at	ribosomal protein S28	*RPS28*	3.0867
208916_at	solute carrier family 1 (neutral amino acid transporter), member 5	*SLC1A5*	0.3943
208922_s_at	nuclear RNA export factor 1	*NXF1*	0.4596
208924_at	ring finger protein 11	*RNF11*	0.4535
208925_at	claudin domain containing 1	*CLDND1*	0.4431
208952_s_at	La ribonucleoprotein domain family, member 4B	*LARP4B*	0.3382
208954_s_at	La ribonucleoprotein domain family, member 4B	*LARP4B*	0.4007
208961_s_at	Kruppel-like factor 6	*KLF6*	2.5039
208962_s_at	fatty acid desaturase 1	*FADS1*	1.7655
208963_x_at	fatty acid desaturase 1	*FADS1*	1.5331
208964_s_at	fatty acid desaturase 1	*FADS1*	1.5261
208979_at	nuclear receptor coactivator 6	*NCOA6*	0.4422
208995_s_at	peptidylprolyl isomerase G (cyclophilin G)	*PPIG*	0.4280
209004_s_at	F-box and leucine-rich repeat protein 5	*FBXL5*	0.3434
209007_s_at	chromosome 1 open reading frame 63	*C1orf63*	3.3283
209027_s_at	abl-interactor 1	*ABI1*	0.3906
209034_at	proline-rich nuclear receptor coactivator 1	*PNRC1*	0.4925
209055_s_at	CDC5 cell division cycle 5-like (S. pombe)	*CDC5L*	0.4796
209067_s_at	heterogeneous nuclear ribonucleoprotein D-like	*HNRPDL*	1.5012
209068_at	heterogeneous nuclear ribonucleoprotein D-like	*HNRPDL*	2.7055
209085_x_at	replication factor C (activator 1) 1, 145 kDa	*RFC1*	0.4968
209091_s_at	SH3-domain GRB2-like endophilin B1	*SH3GLB1*	0.4253
209099_x_at	jagged 1 (Alagille syndrome)	*JAG1*	0.3782
209102_s_at	HMG-box transcription factor 1	*HBP1*	1.8251
209112_at	cyclin-dependent kinase inhibitor 1B (p27, Kip1)	*CDKN1B*	0.4690
209115_at	ubiquitin-like modifier activating enzyme 3	*UBA3*	0.4756
209122_at	perilipin 2	*PLIN2*	0.4135
209124_at	myeloid differentiation primary response gene (88)	*MYD88*	0.3917

Table S1. *Cont.*

Probe Set ID	Gene Title	Gene Symbol	ADE_ave/Corresponding DMSO
209136_s_at	ubiquitin specific peptidase 10	*USP10*	0.3886
209137_s_at	ubiquitin specific peptidase 10	*USP10*	0.3893
209161_at	PRP4 pre-mRNA processing factor 4 homolog (yeast)	*PRPF4*	0.2924
209162_s_at	PRP4 pre-mRNA processing factor 4 homolog (yeast)	*PRPF4*	0.3220
209174_s_at	glutamine-rich 1	*QRICH1*	0.4915
209199_s_at	myocyte enhancer factor 2C	*MEF2C*	1.5226
209222_s_at	oxysterol binding protein-like 2	*OSBPL2*	0.3415
209240_at	*O*-linked *N*-acetylglucosamine (GlcNAc) transferase	*OGT*	1.7389
209257_s_at	structural maintenance of chromosomes 3	*SMC3*	0.4894
209258_s_at	structural maintenance of chromosomes 3	*SMC3*	0.4500
209259_s_at	structural maintenance of chromosomes 3	*SMC3*	0.4724
209262_s_at	nuclear receptor subfamily 2, group F, member 6	*NR2F6*	0.3666
209271_at	bromodomain PHD finger transcription factor	*BPTF*	0.2290
209273_s_at	iron-sulfur cluster assembly 1 homolog (S. cerevisiae)	*ISCA1*	0.4803
209274_s_at	iron-sulfur cluster assembly 1 homolog (S. cerevisiae)	*ISCA1*	0.4763
209282_at	protein kinase D2	*PRKD2*	0.4624
209284_s_at	chromosome 3 open reading frame 63	*C3orf63*	0.4958
209287_s_at	CDC42 effector protein (Rho GTPase binding) 3	*CDC42EP3*	0.4343
209296_at	protein phosphatase 1B (formerly 2C), magnesium-dependent, beta isoform	*PPM1B*	0.1153
209304_x_at	growth arrest and DNA-damage-inducible, beta	*GADD45B*	1.7149
209305_s_at	growth arrest and DNA-damage-inducible, beta	*GADD45B*	1.6447
209336_at	PWP2 periodic tryptophan protein homolog (yeast)	*PWP2*	0.3520
209357_at	Cbp/p300-interacting transactivator, with Glu/Asp-rich carboxy-terminal domain, 2	*CITED2*	0.4512
209358_at	TAF11 RNA polymerase II, TATA box binding protein (TBP)-associated factor, 28 kDa	*TAF11*	0.3282
209375_at	xeroderma pigmentosum, complementation group C	*XPC*	0.3108
209382_at	polymerase (RNA) III (DNA directed) polypeptide C (62kD)	*POLR3C*	0.3286
209398_at	histone cluster 1, H1c	*HIST1H1C*	1.6145
209406_at	BCL2-associated athanogene 2	*BAG2*	0.3574

Table S1. *Cont.*

Probe Set ID	Gene Title	Gene Symbol	ADE_ave/Corresponding DMSO
209427_at	smoothelin	*SMTN*	0.3869
209428_s_at	zinc finger protein-like 1	*ZFPL1*	0.4666
209431_s_at	POZ (BTB) and AT hook containing zinc finger 1	*PATZ1*	0.4365
209435_s_at	Rho/Rac guanine nucleotide exchange factor (GEF) 2	*ARHGEF2*	0.2864
209446_s_at	chromosome 7 open reading frame 44	*C7orf44*	1.8668
209451_at	TRAF family member-associated NFKB activator	*TANK*	0.4850
209457_at	dual specificity phosphatase 5	*DUSP5*	0.4543
209473_at	ectonucleoside triphosphate diphosphohydrolase 1	*ENTPD1*	1.6226
209474_s_at	ectonucleoside triphosphate diphosphohydrolase 1	*ENTPD1*	1.5613
209484_s_at	NSL1, MIND kinetochore complex component, homolog (S. cerevisiae)	*NSL1*	0.3850
209486_at	UTP3, small subunit (SSU) processome component, homolog (S. cerevisiae)	*UTP3*	0.3700
209494_s_at	POZ (BTB) and AT hook containing zinc finger 1	*PATZ1*	0.3433
209517_s_at	ash2 (absent, small, or homeotic)-like (Drosophila)	*ASH2L*	0.4114
209527_at	exosome component 2	*EXOSC2*	0.4954
209533_s_at	phospholipase A2-activating protein	*PLAA*	0.3745
209565_at	ring finger protein 113A	*RNF113A*	0.4636
209567_at	RRS1 ribosome biogenesis regulator homolog (S. cerevisiae)	*RRS1*	0.3010
209572_s_at	embryonic ectoderm development	*EED*	0.3066
209579_s_at	methyl-CpG binding domain protein 4	*MBD4*	0.4907
209580_s_at	methyl-CpG binding domain protein 4	*MBD4*	0.2772
209585_s_at	multiple inositol polyphosphate histidine phosphatase, 1	*MINPP1*	0.4431
209586_s_at	prune homolog (Drosophila)	*PRUNE*	0.3012
209593_s_at	torsin family 1, member B (torsin B)	*TOR1B*	0.3745
209606_at	cytohesin 1 interacting protein	*CYTIP*	0.3968
209636_at	nuclear factor of kappa light polypeptide gene enhancer in B-cells 2 (p49/p100)	*NFKB2*	0.3945
209642_at	budding uninhibited by benzimidazoles 1 homolog (yeast)	*BUB1*	0.3996

Table S1. *Cont.*

**Probe Set ID**	**Gene Title**	**Gene Symbol**	**ADE_ave/Corresponding DMSO**
209644_x_at	cyclin-dependent kinase inhibitor 2A (melanoma, p16, inhibits CDK4)	*CDKN2A*	1.5256
209654_at	KIAA0947	*KIAA0947*	0.4461
209658_at	cell division cycle 16 homolog (S. cerevisiae)	*CDC16*	0.4877
209659_s_at	cell division cycle 16 homolog (S. cerevisiae)	*CDC16*	0.4903
209662_at	centrin, EF-hand protein, 3 (CDC31 homolog, yeast)	*CETN3*	0.4078
209670_at	T cell receptor alpha constant	*TRAC*	1.7473
209671_x_at	T cell receptor alpha locus/T cell receptor alpha constant	*TRA@/TRAC*	2.1949
209680_s_at	kinesin family member C1	*KIFC1*	0.2191
209688_s_at	coiled-coil domain containing 93	*CCDC93*	0.4408
209695_at	protein tyrosine phosphatase type IVA, member 3	*PTP4A3*	1.6314
209748_at	spastin	*SPAST*	0.3865
209760_at	KIAA0922	*KIAA0922*	0.4269
209798_at	nuclear protein, ataxia-telangiectasia locus	*NPAT*	0.2526
209799_at	protein kinase, AMP-activated, alpha 1 catalytic subunit	*PRKAA1*	0.4050
209814_at	zinc finger protein 330	*ZNF330*	0.4020
209824_s_at	aryl hydrocarbon receptor nuclear translocator-like	*ARNTL*	0.4255
209827_s_at	interleukin 16 (lymphocyte chemoattractant factor)	*IL16*	0.4482
209845_at	makorin ring finger protein 1	*MKRN1*	1.6318
209849_s_at	RAD51 homolog C (S. cerevisiae)	*RAD51C*	0.3555
209863_s_at	tumor protein p63	*TP63*	1.6137
209891_at	SPC25, NDC80 kinetochore complex component, homolog (S. cerevisiae)	*SPC25*	0.4729
209892_at	fucosyltransferase 4 (alpha (1,3) fucosyltransferase, myeloid-specific)	*FUT4*	0.2444
209893_s_at	fucosyltransferase 4 (alpha (1,3) fucosyltransferase, myeloid-specific)	*FUT4*	0.3789
209916_at	dehydrogenase E1 and transketolase domain containing 1	*DHTKD1*	1.5910
209919_x_at	gamma-glutamyltransferase 1	*GGT1*	1.6504
209921_at	solute carrier family 7, (cationic amino acid transporter, y+ system) member 11	*SLC7A11*	0.4482
209928_s_at	musculin (activated B-cell factor-1)	*MSC*	0.1415
210001_s_at	suppressor of cytokine signaling 1	*SOCS1*	0.2655

Table S1. *Cont.*

Probe Set ID	Gene Title	Gene Symbol	ADE_ave/Corresponding DMSO
210017_at	mucosa associated lymphoid tissue lymphoma translocation gene 1	*MALT1*	0.3405
210018_x_at	mucosa associated lymphoid tissue lymphoma translocation gene 1	*MALT1*	0.4941
210101_x_at	SH3-domain GRB2-like endophilin B1	*SH3GLB1*	0.4023
210212_x_at	mature T-cell proliferation 1 neighbor	*MTCP1NB*	1.7215
210235_s_at	protein tyrosine phosphatase, receptor type, f polypeptide (PTPRF), interacting protein (liprin), alpha 1	*PPFIA1*	0.4411
210252_s_at	MAP-kinase activating death domain	*MADD*	0.4554
210266_s_at	tripartite motif-containing 33	*TRIM33*	0.4851
210279_at	G protein-coupled receptor 18	*GPR18*	0.2244
210356_x_at	membrane-spanning 4-domains, subfamily A, member 1	*MS4A1*	1.5324
210387_at	histone cluster 1, H2bg	*HIST1H2BG*	2.8097
210449_x_at	mitogen-activated protein kinase 14	*MAPK14*	0.4828
210538_s_at	baculoviral IAP repeat-containing 3	*BIRC3*	0.3136
210541_s_at	tripartite motif-containing 27	*TRIM27*	0.2068
210573_s_at	polymerase (RNA) III (DNA directed) polypeptide C (62kD)	*POLR3C*	0.3491
210592_s_at	spermidine/spermine *N*1-acetyltransferase 1	*SAT1*	1.5579
210621_s_at	RAS p21 protein activator (GTPase activating protein) 1	*RASA1*	0.4747
210649_s_at	AT rich interactive domain 1A (SWI-like)	*ARID1A*	0.3646
210681_s_at	ubiquitin specific peptidase 15	*USP15*	0.4692
210779_x_at	survival of motor neuron protein interacting protein 1	*SIP1*	0.4046
210827_s_at	E74-like factor 3 (ets domain transcription factor, epithelial-specific)	*ELF3*	0.3493
210946_at	phosphatidic acid phosphatase type 2A	*PPAP2A*	0.4977
210962_s_at	A kinase (PRKA) anchor protein (yotiao) 9	*AKAP9*	0.3219
210971_s_at	aryl hydrocarbon receptor nuclear translocator-like	*ARNTL*	0.3425
210972_x_at	T cell receptor alpha locus/T cell receptor alpha constant/T cell receptor alpha joining 17/T cell receptor alpha variable 20	*TRA@/TRAC /TRAJ17/TRAV20*	2.0916
210993_s_at	SMAD family member 1	*SMAD1*	0.4584
211061_s_at	mannosyl (alpha-1,6-)-glycoprotein beta-1,2-*N*-acetylglucosaminyltransferase	*MGAT2*	0.1679

Table S1. *Cont.*

Probe Set ID	Gene Title	Gene Symbol	ADE_ave/Corresponding DMSO
211080_s_at	NIMA (never in mitosis gene a)-related kinase 2	*NEK2*	0.3147
211090_s_at	PRP4 pre-mRNA processing factor 4 homolog B (yeast)	*PRPF4B*	0.3853
211114_x_at	survival of motor neuron protein interacting protein 1	*SIP1*	0.4022
211115_x_at	survival of motor neuron protein interacting protein 1	*SIP1*	0.3523
211257_x_at	zinc finger protein 638	*ZNF638*	0.2377
211285_s_at	ubiquitin protein ligase E3A	*UBE3A*	0.4439
211336_x_at	leukocyte immunoglobulin-like receptor, subfamily B (with TM and ITIM domains), member 1	*LILRB1*	1.7621
211383_s_at	WD repeat domain 37	*WDR37*	0.3785
211392_s_at	POZ (BTB) and AT hook containing zinc finger 1	*PATZ1*	0.3865
211423_s_at	sterol-C5-desaturase (ERG3 delta-5-desaturase homolog, S. cerevisiae)-like	*SC5DL*	0.3897
211450_s_at	mutS homolog 6 (E. coli)	*MSH6*	0.4394
211454_x_at	FKSG49	*FKSG49*	1.5925
211530_x_at	major histocompatibility complex, class I, G	*HLA-G*	1.5550
211575_s_at	ubiquitin protein ligase E3A	*UBE3A*	0.3116
211600_at	protein tyrosine phosphatase, receptor type, O	*PTPRO*	1.7699
211654_x_at	major histocompatibility complex, class II, DQ beta 1	*HLA-DQB1*	1.6637
211686_s_at	MAK16 homolog (S. cerevisiae)	*MAK16*	0.2894
211692_s_at	BCL2 binding component 3	*BBC3*	1.6744
211724_x_at	missing oocyte, meiosis regulator, homolog (Drosophila)	*MIOS*	0.3674
211792_s_at	cyclin-dependent kinase inhibitor 2C (p18, inhibits CDK4)	*CDKN2C*	0.4478
211801_x_at	mitofusin 1	*MFN1*	0.4884
211929_at	heterogeneous nuclear ribonucleoprotein A3	*HNRNPA3*	1.5334
211962_s_at	zinc finger protein 36, C3H type-like 1	*ZFP36L1*	0.3687
212000_at	splicing factor, arginine/serine-rich 14	*SFRS14*	1.6157
212001_at	splicing factor, arginine/serine-rich 14	*SFRS14*	2.0654
212020_s_at	antigen identified by monoclonal antibody Ki-67	*MKI67*	0.4805
212022_s_at	antigen identified by monoclonal antibody Ki-67	*MKI67*	0.4600
212023_s_at	antigen identified by monoclonal antibody Ki-67	*MKI67*	0.4016
212056_at	KIAA0182	*KIAA0182*	0.4777
212108_at	Fas associated factor family member 2	*FAF2*	0.4493

Table S1. *Cont.*

Probe Set ID	Gene Title	Gene Symbol	ADE_ave/Corresponding DMSO
212111_at	syntaxin 12	*STX12*	0.3622
212112_s_at	syntaxin 12	*STX12*	0.4096
212116_at	tripartite motif-containing 27	*TRIM27*	0.2156
212132_at	LSM14A, SCD6 homolog A (S. cerevisiae)	*LSM14A*	0.4884
212152_x_at	AT rich interactive domain 1A (SWI-like)	*ARID1A*	0.4672
212168_at	RNA binding motif protein 12	*RBM12*	0.3406
212173_at	adenylate kinase 2	*AK2*	1.5220
212177_at	splicing factor, arginine/serine-rich 18	*SFRS18*	1.7773
212195_at	interleukin 6 signal transducer (gp130, oncostatin M receptor)	*IL6ST*	0.4749
212201_at	ankyrin repeat and LEM domain containing 2	*ANKLE2*	0.4277
212211_at	ankyrin repeat domain 17	*ANKRD17*	0.4645
212222_at	proteasome (prosome, macropain) activator subunit 4	*PSME4*	0.4318
212260_at	GRB10 interacting GYF protein 2	*GIGYF2*	0.3778
212261_at	GRB10 interacting GYF protein 2	*GIGYF2*	0.4736
212264_s_at	wings apart-like homolog (Drosophila)	*WAPAL*	0.3564
212267_at	wings apart-like homolog (Drosophila)	*WAPAL*	0.2625
212269_s_at	minichromosome maintenance complex component 3 associated protein	*MCM3AP*	0.4783
212306_at	cytoplasmic linker associated protein 2	*CLASP2*	0.3299
212313_at	CHMP family, member 7	*CHMP7*	0.4588
212356_at	KIAA0323	*KIAA0323*	0.4439
212361_s_at	ATPase, Ca^{2+} transporting, cardiac muscle, slow twitch 2	*ATP2A2*	0.2242
212373_at	fem-1 homolog b (C. elegans)	*FEM1B*	0.4563
212375_at	E1A binding protein p400	*EP400*	0.4769
212397_at	radixin	*RDX*	0.3813
212398_at	radixin	*RDX*	0.4406
212402_at	zinc finger CCCH-type containing 13	*ZC3H13*	0.4035
212405_s_at	methyltransferase like 13	*METTL13*	0.3179
212407_at	methyltransferase like 13	*METTL13*	0.3783
212418_at	E74-like factor 1 (ets domain transcription factor)	*ELF1*	0.4405
212420_at	E74-like factor 1 (ets domain transcription factor)	*ELF1*	0.4518
212429_s_at	general transcription factor IIIC, polypeptide 2, beta 110 kDa	*GTF3C2*	0.4755
212434_at	GrpE-like 1, mitochondrial (E. coli)	*GRPEL1*	0.4070
212436_at	tripartite motif-containing 33	*TRIM33*	0.3059

Table S1. *Cont.*

Probe Set ID	Gene Title	Gene Symbol	ADE_ave/Corresponding DMSO
212439_at	inositol hexakisphosphate kinase 1	*IP6K1*	0.4803
212441_at	KIAA0232	*KIAA0232*	0.4777
212445_s_at	neural precursor cell expressed, developmentally down-regulated 4-like	*NEDD4L*	0.4875
212453_at	KIAA1279	*KIAA1279*	0.4204
212454_x_at	heterogeneous nuclear ribonucleoprotein D-like	*HNRPDL*	3.7212
212458_at	sprouty-related, EVH1 domain containing 2	*SPRED2*	0.3361
212465_at	SET domain containing 3	*SETD3*	0.4942
212480_at	cytospin A	*CYTSA*	0.4937
212485_at	G patch domain containing 8	*GPATCH8*	0.4150
212496_s_at	lysine (K)-specific demethylase 4B	*KDM4B*	0.4208
212500_at	2-aminoethanethiol (cysteamine) dioxygenase	*ADO*	0.2550
212501_at	CCAAT/enhancer binding protein (C/EBP), beta	*CEBPB*	0.1776
212502_at	2-aminoethanethiol (cysteamine) dioxygenase	*ADO*	0.2663
212506_at	phosphatidylinositol binding clathrin assembly protein	*PICALM*	0.3364
212513_s_at	ubiquitin specific peptidase 33	*USP33*	0.4842
212548_s_at	FRY-like	*FRYL*	0.4100
212557_at	zinc finger protein 451	*ZNF451*	0.4658
212564_at	potassium channel tetramerisation domain containing 2	*KCTD2*	0.4564
212571_at	chromodomain helicase DNA binding protein 8	*CHD8*	0.4755
212589_at	related RAS viral (r-ras) oncogene homolog 2	*RRAS2*	0.4826
212591_at	RNA binding motif protein 34	*RBM34*	0.2913
212596_s_at	HMG box domain containing 4	*HMGXB4*	0.3414
212597_s_at	HMG box domain containing 4	*HMGXB4*	0.2234
212612_at	REST corepressor 1	*RCOR1*	0.3815
212638_s_at	WW domain containing E3 ubiquitin protein ligase 1	*WWP1*	0.4695
212648_at	DEAH (Asp-Glu-Ala-His) box polypeptide 29	*DHX29*	0.4786
212653_s_at	EH domain binding protein 1	*EHBP1*	0.2513
212663_at	FK506 binding protein 15, 133 kDa	*FKBP15*	0.4222
212674_s_at	DEAH (Asp-Glu-Ala-His) box polypeptide 30	*DHX30*	0.3638
212675_s_at	centrosomal protein 68 kDa	*CEP68*	0.3111
212685_s_at	transducin (beta)-like 2	*TBL2*	0.3378
212688_at	phosphoinositide-3-kinase, catalytic, beta polypeptide	*PIK3CB*	0.3653
212711_at	calmodulin regulated spectrin-associated protein 1	*CAMSAP1*	0.4125
212724_at	Rho family GTPase 3	*RND3*	0.4222

Table S1. *Cont.*

Probe Set ID	Gene Title	Gene Symbol	ADE_ave/Corresponding DMSO
212731_at	ankyrin repeat domain 46	*ANKRD46*	0.4150
212740_at	phosphoinositide-3-kinase, regulatory subunit 4	*PIK3R4*	0.4867
212747_at	ankyrin repeat and sterile alpha motif domain containing 1A	*ANKS1A*	0.3838
212749_s_at	ring finger and CHY zinc finger domain containing 1	*RCHY1*	0.4495
212754_s_at	MON2 homolog (S. cerevisiae)	*MON2*	0.4307
212756_s_at	ubiquitin protein ligase E3 component n-recognin 2	*UBR2*	0.4668
212765_at	calmodulin regulated spectrin-associated protein 1-like 1	*CAMSAP1L1*	0.4191
212766_s_at	interferon stimulated exonuclease gene 20 kDa-like 2	*ISG20L2*	0.3768
212781_at	retinoblastoma binding protein 6	*RBBP6*	0.2168
212783_at	retinoblastoma binding protein 6	*RBBP6*	0.3023
212787_at	YLP motif containing 1	*YLPM1*	0.3377
212804_s_at	GTPase activating protein and VPS9 domains 1	*GAPVD1*	0.4425
212812_at	–	–	0.4984
212825_at	PAX interacting (with transcription-activation domain) protein 1	*PAXIP1*	0.3755
212835_at	family with sequence similarity 175, member B	*FAM175B*	0.2857
212837_at	family with sequence similarity 175, member B	*FAM175B*	0.3052
212841_s_at	PTPRF interacting protein, binding protein 2 (liprin beta 2)	*PPFIBP2*	0.4164
212855_at	DCN1, defective in cullin neddylation 1, domain containing 4 (S. cerevisiae)	*DCUN1D4*	0.4196
212861_at	major facilitator superfamily domain containing 5	*MFSD5*	0.3677
212875_s_at	C2 calcium-dependent domain containing 2	*C2CD2*	0.4438
212880_at	WD repeat domain 7	*WDR7*	0.4969
212881_at	protein inhibitor of activated STAT, 4	*PIAS4*	0.2652
212885_at	M-phase phosphoprotein 10 (U3 small nucleolar ribonucleoprotein)	*MPHOSPH10*	0.4549
212893_at	zinc finger, ZZ-type containing 3	*ZZZ3*	0.3506
212894_at	suppressor of var1, 3-like 1 (S. cerevisiae)	*SUPV3L1*	0.4928
212898_at	KIAA0406	*KIAA0406*	0.2954
212899_at	cell division cycle 2-like 6 (CDK8-like)	*CDC2L6*	0.4584
212900_at	SEC24 family, member A (S. cerevisiae)	*SEC24A*	0.4193
212904_at	leucine rich repeat containing 47	*LRRC47*	0.4482

Table S1. *Cont.*

Probe Set ID	Gene Title	Gene Symbol	ADE_ave/Corresponding DMSO
212905_at	cleavage stimulation factor, 3' pre-RNA, subunit 2, 64 kDa, tau variant	*CSTF2T*	0.4776
212910_at	THAP domain containing 11	*THAP11*	0.1698
212920_at	–	–	0.3823
212949_at	non-SMC condensin I complex, subunit H	*NCAPH*	0.3579
212964_at	hypermethylated in cancer 2	*HIC2*	0.4615
212978_at	leucine rich repeat containing 8 family, member B	*LRRC8B*	0.4537
213017_at	abhydrolase domain containing 3	*ABHD3*	0.4260
213019_at	RAN binding protein 6	*RANBP6*	0.1697
213021_at	golgi SNAP receptor complex member 1	*GOSR1*	0.3880
213035_at	ankyrin repeat domain 28	*ANKRD28*	1.8091
213046_at	poly(A) binding protein, nuclear 1	*PABPN1*	3.6114
213049_at	GTPase activating Rap/RanGAP domain-like 1	*GARNL1*	0.2914
213063_at	zinc finger CCCH-type containing 14	*ZC3H14*	0.4465
213064_at	zinc finger CCCH-type containing 14	*ZC3H14*	0.4431
213069_at	HEG homolog 1 (zebrafish)	*HEG1*	0.4326
213070_at	phosphoinositide-3-kinase, class 2, alpha polypeptide	*PIK3C2A*	0.4948
213090_s_at	TAF4 RNA polymerase II, TATA box binding protein (TBP)-associated factor, 135 kDa	*TAF4*	0.3134
213117_at	kelch-like 9 (Drosophila)	*KLHL9*	0.3089
213123_at	microfibrillar-associated protein 3	*MFAP3*	0.4469
213126_at	mediator complex subunit 8	*MED8*	0.3017
213127_s_at	mediator complex subunit 8	*MED8*	0.3692
213128_s_at	ubiquitin protein ligase E3A	*UBE3A*	0.3072
213145_at	F-box and leucine-rich repeat protein 14	*FBXL14*	0.4408
213153_at	SET domain containing 1B	*SETD1B*	0.3378
213184_at	SUMO1/sentrin specific peptidase 5	*SENP5*	0.4881
213189_at	MYC induced nuclear antigen	*MINA*	0.4423
213233_s_at	kelch-like 9 (Drosophila)	*KLHL9*	0.4332
213262_at	spastic ataxia of Charlevoix-Saguenay (sacsin)	*SACS*	0.3587
213286_at	zinc finger RNA binding protein	*ZFR*	0.4637
213291_s_at	ubiquitin protein ligase E3A	*UBE3A*	0.2897
213301_x_at	tripartite motif-containing 24	*TRIM24*	0.3631
213309_at	phospholipase C-like 2	*PLCL2*	0.4622
213346_at	chromosome 13 open reading frame 27	*C13orf27*	0.3533

Table S1. *Cont.*

Probe Set ID	Gene Title	Gene Symbol	ADE_ave/Corresponding DMSO
213359_at	Heterogeneous nuclear ribonucleoprotein D (AU-rich element RNA binding protein 1, 37 kDa)	*HNRNPD*	4.0092
213365_at	exoribonuclease 2	*ERI2*	0.3947
213372_at	progestin and adipoQ receptor family member III	*PAQR3*	0.3985
213373_s_at	caspase 8, apoptosis-related cysteine peptidase	*CASP8*	0.3860
213390_at	zinc finger CCCH-type containing 4	*ZC3H4*	0.3865
213391_at	dpy-19-like 4 (C. elegans)	*DPY19L4*	0.4492
213405_at	RAB22A, member RAS oncogene family	*RAB22A*	0.4772
213407_at	PH domain and leucine rich repeat protein phosphatase 2	*PHLPP2*	0.4240
213452_at	zinc finger protein 184	*ZNF184*	0.4168
213459_at	ribosomal protein L37a	*RPL37A*	1.5011
213517_at	poly(rC) binding protein 2	*PCBP2*	2.6367
213532_at	ADAM metallopeptidase domain 17	*ADAM17*	0.4871
213546_at	hypothetical protein DKFZp586I1420	*DKFZP586I1420*	1.5110
213578_at	bone morphogenetic protein receptor, type IA	*BMPR1A*	0.4611
213593_s_at	transformer 2 alpha homolog (Drosophila)	*TRA2A*	2.3280
213599_at	Opa interacting protein 5	*OIP5*	0.4042
213608_s_at	SRR1 domain containing	*SRRD*	0.2799
213610_s_at	kelch-like 23 (Drosophila)	*KLHL23*	0.4151
213618_at	ArfGAP with RhoGAP domain, ankyrin repeat and PH domain 2	*ARAP2*	0.3856
213620_s_at	intercellular adhesion molecule 2	*ICAM2*	0.4823
213643_s_at	inositol polyphosphate-5-phosphatase, 75 kDa	*INPP5B*	1.6177
213653_at	methyltransferase like 3	*METTL3*	2.2853
213670_x_at	NOL1/NOP2/Sun domain family, member 5B	*NSUN5B*	1.6390
213696_s_at	mediator complex subunit 8	*MED8*	0.3191
213708_s_at	MAX-like protein X	*MLX*	0.2927
213743_at	cyclin T2	*CCNT2*	1.5741
213761_at	Mdm1 nuclear protein homolog (mouse)	*MDM1*	0.4802
213775_x_at	zinc finger protein 638	*ZNF638*	0.3009
213838_at	nucleolar protein 7, 27 kDa	*NOL7*	0.4690
213848_at	dual specificity phosphatase 7	*DUSP7*	0.2787
213872_at	Chromosome 6 open reading frame 62	*C6orf62*	0.4525
213875_x_at	chromosome 6 open reading frame 62	*C6orf62*	0.4804
213918_s_at	Nipped-B homolog (Drosophila)	*NIPBL*	0.2508
213923_at	RAP2B, member of RAS oncogene family	*RAP2B*	0.4947
213926_s_at	ArfGAP with FG repeats 1	*AGFG1*	0.4162

Table S1. *Cont.*

Probe Set ID	Gene Title	Gene Symbol	ADE_ave/Corresponding DMSO
213931_at	inhibitor of DNA binding 2, dominant negative helix-loop-helix protein/inhibitor of DNA binding 2B, dominant negative helix-loop-helix protein (pseudogene)	*ID2/ID2B*	1.8545
213988_s_at	spermidine/spermine *N*1-acetyltransferase 1	*SAT1*	1.7034
214016_s_at	splicing factor proline/glutamine-rich (polypyrimidine tract binding protein associated)	*SFPQ*	2.2671
214047_s_at	methyl-CpG binding domain protein 4	*MBD4*	0.3636
214061_at	WD repeat domain 67	*WDR67*	0.2582
214085_x_at	GLI pathogenesis-related 1	*GLIPR1*	1.9128
214126_at	–	–	0.4304
214129_at	phosphodiesterase 4D interacting protein	*PDE4DIP*	0.3771
214194_at	DIS3 mitotic control homolog (S. cerevisiae)	*DIS3*	1.9357
214280_x_at	heterogeneous nuclear ribonucleoprotein A1	*HNRNPA1*	2.1966
214281_s_at	ring finger and CHY zinc finger domain containing 1	*RCHY1*	0.4488
214290_s_at	histone cluster 2, H2aa3///histone cluster 2, H2aa4	*HIST2H2AA3/HIST2H2AA4*	1.5993
214291_at	similar to ribosomal protein L17///ribosomal protein L17	*LOC729046/RPL17*	10.8583
214299_at	topoisomerase (DNA) III alpha	*TOP3A*	0.4494
214426_x_at	chromatin assembly factor 1, subunit A (p150)	*CHAF1A*	0.3272
214427_at	NOP2 nucleolar protein homolog (yeast)	*NOP2*	0.4167
214429_at	myotubularin related protein 6	*MTMR6*	0.3674
214440_at	*N*-acetyltransferase 1 (arylamine *N*-acetyltransferase)	*NAT1*	0.2942
214507_s_at	exosome component 2	*EXOSC2*	0.4270
214659_x_at	YLP motif containing 1	*YLPM1*	0.3164
214662_at	WD repeat domain 43	*WDR43*	0.4733
214683_s_at	CDC-like kinase 1///peptidylprolyl isomerase (cyclophilin)-like 3	*CLK1/PPIL3*	2.9074
214700_x_at	RAP1 interacting factor homolog (yeast)	*RIF1*	0.2826
214710_s_at	cyclin B1	*CCNB1*	0.4785
214722_at	Notch homolog 2 (Drosophila) *N*-terminal like	*NOTCH2NL*	2.5367
214751_at	zinc finger protein 468	*ZNF468*	0.4612
214766_s_at	AT hook containing transcription factor 1	*AHCTF1*	0.3638
214787_at	DENN/MADD domain containing 4A	*DENND4A*	0.4388
214877_at	CDK5 regulatory subunit associated protein 1-like 1	*CDKAL1*	1.5166

Table S1. *Cont.*

Probe Set ID	Gene Title	Gene Symbol	ADE_ave/Corresponding DMSO
214919_s_at	ANKHD1-EIF4EBP3 readthrough transcript/ eukaryotic translation initiation factor 4E binding protein 3	*ANKHD1-EIF4EBP 3/EIF4EBP3*	0.4981
214943_s_at	RNA binding motif protein 34	*RBM34*	0.2778
214946_x_at	family with sequence similarity 21, member A/family with sequence similarity 21, member B/family with sequence similarity 21, member C/family with sequence similarity 21, member D	*FAM21A/FAM21B/ FAM21C/FAM21D*	0.4293
214989_x_at	–	–	1.5969
215009_s_at	SEC31 homolog A (S. cerevisiae)	*SEC31A*	1.6306
215031_x_at	ring finger protein 126	*RNF126*	0.4688
215073_s_at	nuclear receptor subfamily 2, group F, member 2	*NR2F2*	0.4837
215084_s_at	leucine rich repeat containing 42	*LRRC42*	0.3423
215158_s_at	death effector domain containing	*DEDD*	0.4523
215165_x_at	uridine monophosphate synthetase	*UMPS*	0.3126
215236_s_at	phosphatidylinositol binding clathrin assembly protein	*PICALM*	0.2604
215239_x_at	zinc finger protein 273	*ZNF273*	0.4167
215346_at	CD40 molecule, TNF receptor superfamily member 5	*CD40*	0.4600
215373_x_at	–	–	1.5963
215411_s_at	TRAF3 interacting protein 2	*TRAF3IP2*	0.3487
215493_x_at	butyrophilin, subfamily 2, member A1	*BTN2A1*	0.3577
215504_x_at	–	–	1.7256
215933_s_at	hematopoietically expressed homeobox	*HHEX*	0.2860
215942_s_at	G-2 and S-phase expressed 1	*GTSE1*	0.4005
215948_x_at	zinc finger, MYM-type 5	*ZMYM5*	0.3795
216125_s_at	RAN binding protein 9	*RANBP9*	0.4389
216199_s_at	mitogen-activated protein kinase 4	*MAP3K4*	0.2732
216253_s_at	parvin, beta	*PARVB*	1.5066
216267_s_at	transmembrane protein 115	*TMEM115*	0.3509
216348_at	ribosomal protein S17 pseudogene 5	*RPS17P5*	1.5444
216602_s_at	phenylalanyl-tRNA synthetase, alpha subunit	*FARSA*	0.3805
216713_at	KRIT1, ankyrin repeat containing	*KRIT1*	0.4350
216996_s_at	FAST kinase domains 2	*FASTKD2*	0.3203
217043_s_at	mitofusin 1	*MFN1*	0.4022
217078_s_at	CD300a molecule	*CD300A*	0.3757
217099_s_at	gem (nuclear organelle) associated protein 4	*GEMIN4*	0.1145

Table S1. *Cont.*

Probe Set ID	Gene Title	Gene Symbol	ADE_ave/Corresponding DMSO
217196_s_at	calmodulin regulated spectrin-associated protein 1-like 1	*CAMSAP1L1*	0.3384
217329_x_at	–	–	1.6237
217336_at	ribosomal protein S10/ribosomal protein S10 pseudogene 5/ribosomal protein S10 pseudogene 7	*RPS10/RPS10P5/ RPS10P7*	1.5694
217363_x_at	–	–	1.6752
217418_x_at	membrane-spanning 4-domains, subfamily A, member 1	*MS4A1*	1.5110
217448_s_at	TOX high mobility group box family member 4	*TOX4*	0.4637
217619_x_at	–	–	1.8777
217640_x_at	spindle and kinetochore associated complex subunit 1	*SKA1*	0.3733
217672_x_at	eukaryotic translation initiation factor 1	*EIF1*	1.8972
217678_at	solute carrier family 7, (cationic amino acid transporter, y+ system) member 11	*SLC7A11*	0.4204
217730_at	transmembrane BAX inhibitor motif containing 1	*TMBIM1*	1.9169
217742_s_at	WW domain containing adaptor with coiled-coil	*WAC*	0.4326
217781_s_at	zinc finger protein 106 homolog (mouse)	*ZFP106*	0.4536
217798_at	CCR4-NOT transcription complex, subunit 2	*CNOT2*	0.3794
217843_s_at	mediator complex subunit 4	*MED4*	0.4780
217879_at	cell division cycle 27 homolog (S. cerevisiae)	*CDC27*	0.4841
217880_at	cell division cycle 27 homolog (S. cerevisiae)	*CDC27*	0.4623
217881_s_at	cell division cycle 27 homolog (S. cerevisiae)	*CDC27*	0.4953
217893_s_at	akirin 1	*AKIRIN1*	0.4342
217894_at	potassium channel tetramerisation domain containing 3	*KCTD3*	0.4993
217905_at	chromosome 10 open reading frame 119	*C10orf119*	1.5025
217906_at	kelch domain containing 2	*KLHDC2*	0.4352
217909_s_at	MAX-like protein X	*MLX*	0.4728
217910_x_at	MAX-like protein X	*MLX*	0.2775
217911_s_at	BCL2-associated athanogene 3	*BAG3*	0.3311
217912_at	dihydrouridine synthase 1-like (S. cerevisiae)	*DUS1L*	1.5629
217936_at	Rho GTPase activating protein 5	*ARHGAP5*	0.4830
217941_s_at	erbb2 interacting protein	*ERBB2IP*	0.4582
217950_at	nitric oxide synthase interacting protein	*NOSIP*	0.4658
217954_s_at	PHD finger protein 3	*PHF3*	0.4947
217965_s_at	SAP30 binding protein	*SAP30BP*	0.4323
217976_s_at	dynein, cytoplasmic 1, light intermediate chain 1	*DYNC1LI1*	0.2763

Table S1. *Cont.*

Probe Set ID	Gene Title	Gene Symbol	ADE_ave/Corresponding DMSO
217986_s_at	bromodomain adjacent to zinc finger domain, 1A	*BAZ1A*	0.4687
217995_at	sulfide quinone reductase-like (yeast)	*SQRDL*	1.6723
218013_x_at	dynactin 4 (p62)	*DCTN4*	0.4960
218016_s_at	polymerase (RNA) III (DNA directed) polypeptide E (80kD)	*POLR3E*	0.3873
218023_s_at	family with sequence similarity 53, member C	*FAM53C*	0.3802
218041_x_at	solute carrier family 38, member 2	*SLC38A2*	0.3741
218071_s_at	makorin ring finger protein 2	*MKRN2*	0.4800
218088_s_at	Ras-related GTP binding C	*RRAGC*	0.4705
218089_at	chromosome 20 open reading frame 4	*C20orf4*	0.4941
218092_s_at	ArfGAP with FG repeats 1	*AGFG1*	0.4154
218103_at	FtsJ homolog 3 (E. coli)	*FTSJ3*	0.4789
218104_at	testis expressed 10	*TEX10*	0.3662
218107_at	WD repeat domain 26	*WDR26*	0.2617
218129_s_at	nuclear transcription factor Y, beta	*NFYB*	0.4593
218133_s_at	NIF3 NGG1 interacting factor 3-like 1 (S. pombe)	*NIF3L1*	0.4896
218141_at	ubiquitin-conjugating enzyme E2O	*UBE2O*	0.4432
218145_at	tribbles homolog 3 (Drosophila)	*TRIB3*	0.4837
218191_s_at	LMBR1 domain containing 1	*LMBRD1*	0.4775
218196_at	osteopetrosis associated transmembrane protein 1	*OSTM1*	0.4822
218214_at	chromosome 12 open reading frame 44	*C12orf44*	0.3834
218228_s_at	tankyrase, TRF1-interacting ankyrin-related ADP-ribose polymerase 2	*TNKS2*	0.3640
218229_s_at	pogo transposable element with KRAB domain	*POGK*	0.4650
218231_at	*N*-acetylglucosamine kinase	*NAGK*	0.3121
218238_at	GTP binding protein 4	*GTPBP4*	0.2757
218239_s_at	GTP binding protein 4	*GTPBP4*	0.3568
218241_at	golgi autoantigen, golgin subfamily a, 5	*GOLGA5*	0.2441
218244_at	nucleolar protein 8	*NOL8*	0.4821
218246_at	mitochondrial E3 ubiquitin ligase 1	*MUL1*	0.4581
218251_at	MID1 interacting protein 1 (gastrulation specific G12 homolog (zebrafish))	*MID1IP1*	0.3554
218252_at	cytoskeleton associated protein 2	*CKAP2*	0.3209
218259_at	MKL/myocardin-like 2	*MKL2*	0.4512
218263_s_at	zinc finger, BED-type containing 5	*ZBED5*	0.1549
218276_s_at	salvador homolog 1 (Drosophila)	*SAV1*	0.4930
218304_s_at	oxysterol binding protein-like 11	*OSBPL11*	0.3958

Table S1. *Cont.*

Probe Set ID	Gene Title	Gene Symbol	ADE_ave/Corresponding DMSO
218308_at	transforming, acidic coiled-coil containing protein 3	*TACC3*	0.2966
218325_s_at	death inducer-obliterator 1	*DIDO1*	0.4547
218329_at	PR domain containing 4	*PRDM4*	0.4698
218331_s_at	chromosome 10 open reading frame 18	*C10orf18*	0.3095
218341_at	phosphopantothenoylcysteine synthetase	*PPCS*	0.4582
218344_s_at	REST corepressor 3	*RCOR3*	0.3927
218350_s_at	geminin, DNA replication inhibitor	*GMNN*	0.4145
218355_at	kinesin family member 4A	*KIF4A*	0.3521
218356_at	FtsJ homolog 2 (E. coli)	*FTSJ2*	0.3746
218360_at	RAB22A, member RAS oncogene family	*RAB22A*	0.3187
218376_s_at	microtubule associated monoxygenase, calponin and LIM domain containing 1	*MICAL1*	1.6456
218398_at	mitochondrial ribosomal protein S30	*MRPS30*	0.4106
218401_s_at	zinc finger protein 281	*ZNF281*	0.4002
218405_at	activator of basal transcription 1	*ABT1*	0.4140
218411_s_at	MAP3K12 binding inhibitory protein 1	*MBIP*	0.3698
218423_x_at	vacuolar protein sorting 54 homolog (S. cerevisiae)	*VPS54*	0.4577
218437_s_at	leucine zipper transcription factor-like 1	*LZTFL1*	2.2652
218442_at	tetratricopeptide repeat domain 4	*TTC4*	0.2102
218452_at	SWI/SNF related, matrix associated, actin dependent regulator of chromatin, subfamily a-like 1	*SMARCAL1*	0.3988
218458_at	germ cell-less homolog 1 (Drosophila)	*GMCL1*	0.4651
218462_at	brix domain containing 5	*BXDC5*	0.4344
218464_s_at	chromosome 17 open reading frame 63	*C17orf63*	0.3523
218470_at	tyrosyl-tRNA synthetase 2, mitochondrial	*YARS2*	0.4315
218472_s_at	pelota homolog (Drosophila)	*PELO*	0.3577
218474_s_at	potassium channel tetramerisation domain containing 5	*KCTD5*	0.4958
218492_s_at	THAP domain containing 7	*THAP7*	0.4930
218512_at	WD repeat domain 12	*WDR12*	0.4531
218514_at	chromosome 17 open reading frame 71	*C17orf71*	0.3984
218519_at	solute carrier family 35, member A5	*SLC35A5*	0.4027
218520_at	TANK-binding kinase 1	*TBK1*	0.3940
218528_s_at	ring finger protein 38	*RNF38*	0.4026
218534_s_at	angiogenic factor with G patch and FHA domains 1	*AGGF1*	0.3497
218539_at	F-box protein 34	*FBXO34*	0.4859

Table S1. *Cont.*

Probe Set ID	Gene Title	Gene Symbol	ADE_ave/Corresponding DMSO
218542_at	centrosomal protein 55 kDa	*CEP55*	0.4127
218547_at	dehydrodolichyl diphosphate synthase	*DHDDS*	0.4310
218564_at	ring finger and WD repeat domain 3	*RFWD3*	0.4036
218565_at	chromosome 9 open reading frame 114	*C9orf114*	0.2507
218578_at	cell division cycle 73, Paf1/RNA polymerase II complex component, homolog (S. cerevisiae)	*CDC73*	0.3109
218601_at	up-regulated gene 4	*URG4*	0.2741
218602_s_at	HAUS augmin-like complex, subunit 6	*HAUS6*	0.3327
218604_at	LEM domain containing 3	*LEMD3*	0.3044
218612_s_at	tumor suppressing subtransferable candidate 4	*TSSC4*	0.4161
218614_at	chromosome 12 open reading frame 35	*C12orf35*	0.2489
218619_s_at	suppressor of variegation 3-9 homolog 1 (Drosophila)	*SUV39H1*	0.4161
218626_at	eukaryotic translation initiation factor 4E nuclear import factor 1	*EIF4ENIF1*	0.2853
218646_at	chromosome 4 open reading frame 27	*C4orf27*	0.4743
218647_s_at	yrdC domain containing (E. coli)	*YRDC*	1.5458
218648_at	CREB regulated transcription coactivator 3	*CRTC3*	0.4322
218662_s_at	non-SMC condensin I complex, subunit G	*NCAPG*	0.2763
218663_at	non-SMC condensin I complex, subunit G	*NCAPG*	0.2437
218682_s_at	solute carrier family 4 (anion exchanger), member 1, adaptor protein	*SLC4A1AP*	0.3496
218687_s_at	mucin 13, cell surface associated	*MUC13*	0.4745
218695_at	exosome component 4	*EXOSC4*	0.2496
218708_at	NTF2-like export factor 1	*NXT1*	0.4485
218715_at	UTP6, small subunit (SSU) processome component, homolog (yeast)	*UTP6*	0.4574
218722_s_at	coiled-coil domain containing 51	*CCDC51*	0.4059
218726_at	Holliday junction recognition protein	*HJURP*	0.2644
218728_s_at	cornichon homolog 4 (Drosophila)	*CNIH4*	0.4981
218732_at	peptidyl-tRNA hydrolase 2	*PTRH2*	0.4607
218735_s_at	zinc finger protein 544	*ZNF544*	0.4155
218750_at	TATA box binding protein (TBP)-associated factor, RNA polymerase I, D, 41 kDa	*TAF1D*	6.8154
218758_s_at	ribosomal RNA processing 1 homolog (S. cerevisiae)	*RRP1*	0.4605
218763_at	syntaxin 18	*STX18*	0.2798
218776_s_at	transmembrane protein 62	*TMEM62*	0.3526
218777_at	receptor accessory protein 4	*REEP4*	0.4156

Table S1. *Cont.*

Probe Set ID	Gene Title	Gene Symbol	ADE_ave/Corresponding DMSO
218782_s_at	ATPase family, AAA domain containing 2	*ATAD2*	0.3425
218793_s_at	sex comb on midleg-like 1 (Drosophila)	*SCML1*	1.5424
218819_at	integrator complex subunit 6	*INTS6*	0.2475
218823_s_at	potassium channel tetramerisation domain containing 9	*KCTD9*	0.2941
218837_s_at	ubiquitin-conjugating enzyme E2D 4 (putative)	*UBE2D4*	0.4999
218842_at	RNA polymerase II associated protein 3	*RPAP3*	0.4491
218846_at	mediator complex subunit 23	*MED23*	0.4627
218850_s_at	LIM domains containing 1	*LIMD1*	0.4978
218853_s_at	motile sperm domain containing 1	*MOSPD1*	0.4160
218859_s_at	ESF1, nucleolar pre-rRNA processing protein, homolog (S. cerevisiae)	*ESF1*	0.3903
218873_at	gon-4-like (C. elegans)	*GON4L*	0.4599
218882_s_at	WD repeat domain 3	*WDR3*	0.2898
218887_at	mitochondrial ribosomal protein L2	*MRPL2*	0.4729
218889_at	nucleolar complex associated 3 homolog (S. cerevisiae)	*NOC3L*	0.4819
218898_at	family with sequence similarity 57, member A	*FAM57A*	0.4145
218912_at	GRIP and coiled-coil domain containing 1	*GCC1*	0.2433
218917_s_at	AT rich interactive domain 1A (SWI-like)	*ARID1A*	0.3374
218929_at	CDKN2A interacting protein	*CDKN2AIP*	1.9541
218932_at	zinc finger, HIT type 6	*ZNHIT6*	0.4880
218933_at	spermatogenesis associated 5-like 1	*SPATA5L1*	0.3211
218936_s_at	coiled-coil domain containing 59	*CCDC59*	0.3828
218937_at	zinc finger protein 434	*ZNF434*	0.4493
218949_s_at	glutaminyl-tRNA synthase (glutamine-hydrolyzing)-like 1	*QRSL1*	0.4362
218954_s_at	BRF2, subunit of RNA polymerase III transcription initiation factor, BRF1-like	*BRF2*	0.4137
218956_s_at	ATP synthase, H^+ transporting, mitochondrial F0 complex, subunit F2/pentatricopeptide repeat domain 1	*ATP5J2/PTCD1*	0.3065
218968_s_at	zinc finger protein 64 homolog (mouse)	*ZFP64*	0.3632
218975_at	collagen, type V, alpha 3	*COL5A3*	1.9309
218979_at	RMI1, RecQ mediated genome instability 1, homolog (S. cerevisiae)	*RMI1*	0.4321
218989_x_at	solute carrier family 30 (zinc transporter), member 5	*SLC30A5*	0.4995
218993_at	RNA methyltransferase like 1	*RNMTL1*	0.3736
219002_at	FAST kinase domains 1	*FASTKD1*	0.4351

Table S1. *Cont.*

**Probe Set ID**	**Gene Title**	**Gene Symbol**	**ADE_ave/Corresponding DMSO**
219006_at	NADH dehydrogenase (ubiquinone) 1 alpha subcomplex, assembly factor 4	*NDUFAF4*	0.3950
219035_s_at	ring finger protein 34	*RNF34*	0.4554
219037_at	ribosomal RNA processing 15 homolog (S. cerevisiae)	*RRP15*	0.4922
219053_s_at	vacuolar protein sorting 37 homolog C (S. cerevisiae)	*VPS37C*	0.4707
219068_x_at	ATPase family, AAA domain containing 3A	*ATAD3A*	1.5119
219074_at	transmembrane protein 184C	*TMEM184C*	0.3334
219083_at	SHQ1 homolog (S. cerevisiae)	*SHQ1*	0.3460
219089_s_at	zinc finger protein 576	*ZNF576*	0.4950
219094_at	armadillo repeat containing 8	*ARMC8*	1.5443
219099_at	chromosome 12 open reading frame 5	*C12orf5*	0.3527
219120_at	chromosome 2 open reading frame 44	*C2orf44*	0.3376
219124_at	chromosome 8 open reading frame 41	*C8orf41*	0.2379
219129_s_at	SAP30-like	*SAP30L*	0.4656
219131_at	UbiA prenyltransferase domain containing 1	*UBIAD1*	0.4726
219133_at	3-oxoacyl-ACP synthase, mitochondrial	*OXSM*	0.2486
219141_s_at	autophagy/beclin-1 regulator 1	*AMBRA1*	0.3719
219146_at	chromosome 17 open reading frame 42	*C17orf42*	0.3709
219149_x_at	debranching enzyme homolog 1 (S. cerevisiae)	*DBR1*	0.2660
219166_at	chromosome 14 open reading frame 104	*C14orf104*	0.2330
219169_s_at	transcription factor B1, mitochondrial	*TFB1M*	0.4230
219176_at	chromosome 2 open reading frame 47	*C2orf47*	0.4093
219177_at	brix domain containing 2	*BXDC2*	0.2745
219200_at	FAST kinase domains 3	*FASTKD3*	0.4598
219212_at	heat shock 70 kDa protein 14	*HSPA14*	0.4119
219226_at	Cdc2-related kinase, arginine/serine-rich	*CRKRS*	0.4183
219231_at	trimethylguanosine synthase homolog (S. cerevisiae)	*TGS1*	0.2814
219238_at	phosphatidylinositol glycan anchor biosynthesis, class V	*PIGV*	0.4570
219244_s_at	mitochondrial ribosomal protein L46	*MRPL46*	0.2915
219253_at	transmembrane protein 185B (pseudogene)	*TMEM185B*	0.4733
219258_at	TIMELESS interacting protein	*TIPIN*	0.3193
219261_at	chromosome 7 open reading frame 26	*C7orf26*	0.2648
219276_x_at	chromosome 9 open reading frame 82	*C9orf82*	0.4413
219286_s_at	RNA binding motif protein 15	*RBM15*	0.3621
219296_at	zinc finger, DHHC-type containing 13	*ZDHHC13*	0.4559

Table S1. *Cont.*

Probe Set ID	Gene Title	Gene Symbol	ADE_ave/Corresponding DMSO
219321_at	membrane protein, palmitoylated 5 (MAGUK p55 subfamily member 5)	*MPP5*	0.3634
219322_s_at	WD repeat domain 8	*WDR8*	0.2845
219328_at	DEAD (Asp-Glu-Ala-Asp) box polypeptide 31	*DDX31*	0.4878
219343_at	cell division cycle 37 homolog (S. cerevisiae)-like 1	*CDC37L1*	0.4838
219353_at	NHL repeat containing 2	*NHLRC2*	0.4431
219420_s_at	chromosome 1 open reading frame 163	*C1orf163*	0.4056
219433_at	BCL6 co-repressor	*BCOR*	0.4976
219439_at	core 1 synthase, glycoprotein-*N*-acetylgalactosamine 3-beta-galactosyltransferase, 1	*C1GALT1*	0.2997
219446_at	resistance to inhibitors of cholinesterase 8 homolog B (C. elegans)	*RIC8B*	0.4257
219459_at	polymerase (RNA) III (DNA directed) polypeptide B	*POLR3B*	0.2239
219460_s_at	transmembrane protein 127	*TMEM127*	0.4847
219467_at	gypsy retrotransposon integrase 1	*GIN1*	0.4355
219486_at	dihydrouridine synthase 2-like, SMM1 homolog (S. cerevisiae)	*DUS2L*	0.3491
219492_at	cysteine-rich hydrophobic domain 2	*CHIC2*	0.1870
219494_at	RAD54 homolog B (S. cerevisiae)	*RAD54B*	0.3937
219502_at	nei endonuclease VIII-like 3 (E. coli)	*NEIL3*	0.3179
219512_at	DSN1, MIND kinetochore complex component, homolog (S. cerevisiae)	*DSN1*	0.4496
219522_at	four jointed box 1 (Drosophila)	*FJX1*	0.4767
219530_at	partner and localizer of BRCA2	*PALB2*	0.3512
219541_at	Lck interacting transmembrane adaptor 1	*LIME1*	1.5240
219544_at	chromosome 13 open reading frame 34	*C13orf34*	0.4483
219551_at	ELL associated factor 2	*EAF2*	0.4522
219565_at	cytochrome P450, family 20, subfamily A, polypeptide 1	*CYP20A1*	0.4158
219575_s_at	component of oligomeric golgi complex 8/// peptide deformylase (mitochondrial)	*COG8/PDF*	0.3377
219577_s_at	ATP-binding cassette, sub-family A (ABC1), member 7	*ABCA7*	1.6990
219581_at	tRNA splicing endonuclease 2 homolog (S. cerevisiae)	*TSEN2*	0.4815
219595_at	similar to zinc finger protein 26 (KOX 20)///zinc finger protein 26	*LOC100287515/ ZNF26*	1.6049

Table S1. *Cont.*

Probe Set ID	Gene Title	Gene Symbol	ADE_ave/Corresponding DMSO
219640_at	claudin 15	*CLDN15*	1.7002
219662_at	chromosome 2 open reading frame 49	*C2orf49*	0.3197
219679_s_at	WW domain containing adaptor with coiled-coil	*WAC*	0.4837
219707_at	copine VII	*CPNE7*	1.6723
219731_at	weakly similar to zinc finger protein 195	*FLJ34077*	0.4269
219733_s_at	solute carrier family 27 (fatty acid transporter), member 5	*SLC27A5*	1.5024
219753_at	stromal antigen 3	*STAG3*	1.5460
219787_s_at	epithelial cell transforming sequence 2 oncogene	*ECT2*	0.3472
219798_s_at	methylphosphate capping enzyme	*MEPCE*	0.2431
219816_s_at	RNA binding motif protein 23	*RBM23*	0.4890
219918_s_at	asp (abnormal spindle) homolog, microcephaly associated (Drosophila)	*ASPM*	0.2807
219920_s_at	GDP-mannose pyrophosphorylase B	*GMPPB*	1.5074
219966_x_at	BTG3 associated nuclear protein	*BANP*	0.2767
219971_at	interleukin 21 receptor	*IL21R*	0.3546
219981_x_at	zinc finger protein 587	*ZNF587*	0.4436
220035_at	nucleoporin 210 kDa	*NUP210*	1.8905
220046_s_at	cyclin L1	*CCNL1*	2.7922
220104_at	zinc finger CCCH-type, antiviral 1	*ZC3HAV1*	1.9500
220235_s_at	chromosome 1 open reading frame 103	*C1orf103*	0.2879
220253_s_at	low density lipoprotein-related protein 12	*LRP12*	0.4186
220295_x_at	DEP domain containing 1	*DEPDC1*	0.3436
220319_s_at	myosin regulatory light chain interacting protein	*MYLIP*	2.9232
220330_s_at	SAM domain, SH3 domain and nuclear localization signals 1	*SAMSN1*	0.4535
220367_s_at	Sin3A-associated protein, 130 kDa	*SAP130*	0.3837
220485_s_at	signal-regulatory protein gamma	*SIRPG*	1.5937
220651_s_at	minichromosome maintenance complex component 10	*MCM10*	0.2639
220742_s_at	*N*-glycanase 1	*NGLY1*	0.3378
220924_s_at	solute carrier family 38, member 2	*SLC38A2*	0.3783
220926_s_at	ER degradation enhancer, mannosidase alpha-like 3	*EDEM3*	0.4270
220934_s_at	transmembrane protein 223	*TMEM223*	0.3533
220937_s_at	ST6 alpha-*N*-acetyl-neuraminyl-2,3-beta-galactosyl-1,3)-*N*-acetylgalactosaminide alpha-2,6-sialyltransferase 4	*ST6GALNAC4*	1.6597
220949_s_at	chromosome 7 open reading frame 49	*C7orf49*	0.3213

Table S1. *Cont.*

Probe Set ID	Gene Title	Gene Symbol	ADE_ave/Corresponding DMSO
220964_s_at	RAB1B, member RAS oncogene family	*RAB1B*	1.5938
221007_s_at	FIP1 like 1 (S. cerevisiae)	*FIP1L1*	0.4228
221020_s_at	solute carrier family 25, member 32	*SLC25A32*	0.3938
221122_at	HRAS-like suppressor 2	*HRASLS2*	1.6261
221190_s_at	chromosome 18 open reading frame 8	*C18orf8*	0.4621
221193_s_at	zinc finger, CCHC domain containing 10	*ZCCHC10*	0.3544
221229_s_at	tRNA methyltransferase 61 homolog B (S. cerevisiae)	*TRMT61B*	0.3567
221257_x_at	F-box protein 38	*FBXO38*	0.4407
221258_s_at	kinesin family member 18A	*KIF18A*	0.2360
221277_s_at	pseudouridylate synthase 3	*PUS3*	0.4712
221286_s_at	hypothetical protein MGC29506	*MGC29506*	1.5510
221311_x_at	LYR motif containing 2	*LYRM2*	0.3475
221480_at	heterogeneous nuclear ribonucleoprotein D (AU-rich element RNA binding protein 1, 37 kDa)	*HNRNPD*	1.5003
221497_x_at	egl nine homolog 1 (C. elegans)	*EGLN1*	0.3536
221518_s_at	ubiquitin specific peptidase 47	*USP47*	0.4135
221520_s_at	cell division cycle associated 8	*CDCA8*	0.4572
221528_s_at	engulfment and cell motility 2	*ELMO2*	0.4304
221536_s_at	large subunit GTPase 1 homolog (S. cerevisiae)	*LSG1*	0.4939
221547_at	PRP18 pre-mRNA processing factor 18 homolog (S. cerevisiae)	*PRPF18*	0.4500
221549_at	glutamate-rich WD repeat containing 1	*GRWD1*	0.3243
221552_at	abhydrolase domain containing 6	*ABHD6*	0.2271
221563_at	dual specificity phosphatase 10	*DUSP10*	0.4966
221571_at	TNF receptor-associated factor 3	*TRAF3*	0.4973
221586_s_at	E2F transcription factor 5, p130-binding	*E2F5*	0.4568
221593_s_at	ribosomal protein L31	*RPL31*	3.1404
221649_s_at	peter pan homolog (Drosophila)	*PPAN*	0.1407
221657_s_at	ankyrin repeat and SOCS box-containing 6	*ASB6*	0.3319
221658_s_at	interleukin 21 receptor	*IL21R*	0.3316
221679_s_at	abhydrolase domain containing 6	*ABHD6*	0.3234
221688_s_at	IMP3, U3 small nucleolar ribonucleoprotein, homolog (yeast)	*IMP3*	0.2570
221706_s_at	unconventional SNARE in the ER 1 homolog (S. cerevisiae)	*USE1*	0.4624
221741_s_at	YTH domain family, member 1	*YTHDF1*	0.3452
221751_at	Solute carrier family 2 (facilitated glucose transporter), member 3 pseudogene 1	*SLC2A3P1*	0.4337

Table S1. *Cont.*

**Probe Set ID**	**Gene Title**	**Gene Symbol**	**ADE_ave/Corresponding DMSO**
221758_at	armadillo repeat containing 6	*ARMC6*	1.7673
221768_at	Splicing factor proline/glutamine-rich (polypyrimidine tract binding protein associated)	*SFPQ*	3.4452
221772_s_at	protein phosphatase 2, regulatory subunit B, delta isoform	*PPP2R2D*	0.3882
221804_s_at	family with sequence similarity 45, member A/ family with sequence similarity 45, member A pseudogene	*FAM45A/FAM45B*	0.4349
221808_at	RAB9A, member RAS oncogene family	*RAB9A*	0.3243
221813_at	F-box protein 42	*FBXO42*	0.3475
221817_at	dolichyl pyrophosphate phosphatase 1	*DOLPP1*	0.4425
221823_at	chromosome 5 open reading frame 30	*C5orf30*	0.3585
221825_at	angel homolog 2 (Drosophila)	*ANGEL2*	0.4710
221848_at	zinc finger, CCCH-type with G patch domain	*ZGPAT*	0.3737
221855_at	succinate dehydrogenase complex assembly factor 1	*SDHAF1*	0.4928
221860_at	heterogeneous nuclear ribonucleoprotein L	*HNRNPL*	4.4629
221890_at	zinc finger protein 335	*ZNF335*	0.4138
221918_at	PCTAIRE protein kinase 2	*PCTK2*	0.3562
221922_at	G-protein signaling modulator 2 (AGS3-like, C. elegans)	*GPSM2*	0.2972
221937_at	synergin, gamma	*SYNRG*	0.4075
221940_at	RNA pseudouridylate synthase domain containing 2	*RPUSD2*	0.3840
221942_s_at	guanylate cyclase 1, soluble, alpha 3	*GUCY1A3*	0.3812
221970_s_at	nucleolar protein 11	*NOL11*	0.3515
221989_at	ribosomal protein L10	*RPL10*	4.1064
222000_at	chromosome 1 open reading frame 174	*C1orf174*	0.3803
222018_at	nascent polypeptide-associated complex alpha subunit/nascent-polypeptide-associated complex alpha polypeptide pseudogene 1	*NACA/NACAP1*	14.4151
222028_at	zinc finger protein 45	*ZNF45*	0.3808
222030_at	SIVA1, apoptosis-inducing factor	*SIVA1*	3.5677
222034_at	Guanine nucleotide binding protein (G protein), beta polypeptide 2-like 1	*GNB2L1*	8.4318
222040_at	heterogeneous nuclear ribonucleoprotein A1	*HNRNPA1*	4.1086
222044_at	PDX1 C-terminal inhibiting factor 1	*PCIF1*	1.6084
222045_s_at	PDX1 C-terminal inhibiting factor 1	*PCIF1*	1.6823

Table S1. *Cont.*

Probe Set ID	Gene Title	Gene Symbol	ADE_ave/Corresponding DMSO
222067_x_at	histone cluster 1, H2bd	*HIST1H2BD*	1.5425
222103_at	activating transcription factor 1	*ATF1*	0.4888
222118_at	centromere protein N	*CENPN*	0.4874
222119_s_at	F-box protein 11	*FBXO11*	0.4165
222130_s_at	FtsJ homolog 2 (E. coli)	*FTSJ2*	0.4062
222163_s_at	spermatogenesis associated 5-like 1	*SPATA5L1*	0.3379
222165_x_at	chromosome 9 open reading frame 16	*C9orf16*	1.7473
222182_s_at	CCR4-NOT transcription complex, subunit 2	*CNOT2*	0.3781
222199_s_at	bridging integrator 3	*BIN3*	0.3904
222201_s_at	caspase 8 associated protein 2	*CASP8AP2*	0.4551
222203_s_at	retinol dehydrogenase 14 (all-trans/9-cis/11-cis)	*RDH14*	0.3862
222239_s_at	integrator complex subunit 6	*INTS6*	0.2361
222243_s_at	transducer of ERBB2, 2	*TOB2*	0.3560
222250_s_at	integrator complex subunit 7	*INTS7*	0.3006
222251_s_at	glucocorticoid modulatory element binding protein 2	*GMEB2*	0.4407
222294_s_at	–	–	1.7564
222311_s_at	splicing factor, arginine/serine-rich 15	*SFRS15*	1.9966
31826_at	FK506 binding protein 15, 133 kDa	*FKBP15*	0.3552
31845_at	E74-like factor 4 (ets domain transcription factor)	*ELF4*	0.3654
32032_at	DiGeorge syndrome critical region gene 14	*DGCR14*	0.4587
32069_at	NEDD4 binding protein 1	*N4BP1*	0.2485
32541_at	protein phosphatase 3 (formerly 2B), catalytic subunit, gamma isoform	*PPP3CC*	0.4408
32723_at	cleavage stimulation factor, 3' pre-RNA, subunit 1, 50 kDa	*CSTF1*	0.3728
34031_i_at	KRIT1, ankyrin repeat containing	*KRIT1*	0.4720
34225_at	Wolf-Hirschhorn syndrome candidate 2	*WHSC2*	0.4894
34689_at	three prime repair exonuclease 1	*TREX1*	0.4046
35254_at	TRAF-type zinc finger domain containing 1	*TRAFD1*	0.4147
35974_at	lymphoid-restricted membrane protein	*LRMP*	1.6055
37028_at	protein phosphatase 1, regulatory (inhibitor) subunit 15A	*PPP1R15A*	1.7214
38157_at	dom-3 homolog Z (C. elegans)	*DOM3Z*	1.5789
38398_at	MAP-kinase activating death domain	*MADD*	0.4570
40562_at	guanine nucleotide binding protein (G protein), alpha 11 (Gq class)	*GNA11*	0.4928
44146_at	glucocorticoid modulatory element binding protein 2	*GMEB2*	0.4711

Table S1. *Cont.*

Probe Set ID	Gene Title	Gene Symbol	ADE_ave/Corresponding DMSO
44669_at	succinate dehydrogenase complex assembly factor 1	*SDHAF1*	0.4631
44783_s_at	hairy/enhancer-of-split related with YRPW motif 1	*HEY1*	0.3914
45288_at	abhydrolase domain containing 6	*ABHD6*	0.3247
46167_at	tetratricopeptide repeat domain 4	*TTC4*	0.2003
46665_at	sema domain, immunoglobulin domain (Ig), transmembrane domain (TM) and short cytoplasmic domain, (semaphorin) 4C	*SEMA4C*	0.4710
47083_at	chromosome 7 open reading frame 26	*C7orf26*	0.3791
47105_at	dihydrouridine synthase 2-like, SMM1 homolog (S. cerevisiae)	*DUS2L*	0.4074
47773_at	F-box protein 42	*FBXO42*	0.4573
49485_at	PR domain containing 4	*PRDM4*	0.4940
51146_at	phosphatidylinositol glycan anchor biosynthesis, class V	*PIGV*	0.3683
51176_at	mediator complex subunit 27	*MED27*	0.3524
52731_at	autophagy/beclin-1 regulator 1	*AMBRA1*	0.4066
53968_at	integrator complex subunit 5	*INTS5*	0.1323
53987_at	RAN binding protein 10	*RANBP10*	0.4756
55065_at	MAP/microtubule affinity-regulating kinase 4	*MARK4*	0.4114
57532_at	dishevelled, dsh homolog 2 (Drosophila)	*DVL2*	0.4147
57539_at	zinc finger, CCCH-type with G patch domain	*ZGPAT*	0.4107
58696_at	exosome component 4	*EXOSC4*	0.2509
59999_at	hypoxia inducible factor 1, alpha subunit inhibitor	*HIF1AN*	0.4728
63009_at	SHQ1 homolog (S. cerevisiae)	*SHQ1*	0.3676
64371_at	splicing factor, arginine/serine-rich 14	*SFRS14*	2.0881
64474_g_at	DiGeorge syndrome critical region gene 8	*DGCR8*	0.4713
91684_g_at	exosome component 4	*EXOSC4*	0.2755
91952_at	DDB1 and CUL4 associated factor 15	*DCAF15*	0.4751
